# Synthesis of Lithium Manganese Oxide and Ti‐Substituted LMO Sorbents for Lithium Extraction in a Spray‐Drying Process

**DOI:** 10.1002/cssc.202500530

**Published:** 2025-05-23

**Authors:** Laura Herrmann, Nicole Bohn, Alisa Pfau, Thomas Kölbel, Helmut Ehrenberg, Joachim R. Binder, Fabian Jeschull

**Affiliations:** ^1^ Institute for Applied Materials (IAM) Karlsruhe Institute of Technology Hermann‐von‐Helmholtz‐Platz 1 D‐76344 Eggenstein‐Leopoldshafen Germany; ^2^ R&D Department EnBW Energie Baden‐Württemberg AG Durlacher Allee 93 76131 Karlsruhe Germany

**Keywords:** geothermal brines, lithium extractions, lithium manganese oxides, lithium recovery, spray‐drying

## Abstract

Lithium manganese oxides (LMO) are highly promising sorbents for lithium extraction from Li
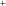
‐containing brines with high salt contents due to their high sorption capacity and high selectivity toward lithium. However, conventional synthesis routes are limited in scale. Therefore, a novel spray‐drying method is presented herein, enabling a scalable synthesis of LMO sorbents for Li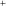 extraction. The ion‐exchange material is studied in both synthetic LiCl solutions and two different geothermal brines from the Upper Rhine Valley, demonstrating improved Li selectivity and extraction capabilities compared to materials from hydrothermal synthesis approaches. The extraction behavior in relevant mildly acidic environments is studied in detail. Further material improvements are achieved by substituting a fraction of Mn by Ti, which greatly reduced the dissolution of manganese during acid treatment in the first 5 extraction cycles from 5.6% to only 1.8%. In addition, the maximum sorption capacity of the Ti‐substituted LMO (LMTO) can be further increased from 5.05 mmol g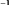 for LMO (35.1 mg g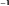) to 5.66 mmol g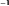 for LMTO (39.3 mg g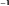) under optimized m/V ratios. Hence, the results reported herein present a pathway toward LMO‐based ion‐exchange materials for the direct lithium extraction on an industrial scale.

## Graphical abstract

Herein, a novel synthetic, scalable approach brines is presented to produce Li_1.6_Mn_1.6_O_4_ (lithium manganese oxides [LMO]) with high phase purity for direct lithium extraction applications from Li‐rich geothermal. Compared to conventional hydrothermal approaches, the LMO materials prepared herein display lower manganese leaching rates, higher extraction capacities, and faster ion‐exchange kinetics.

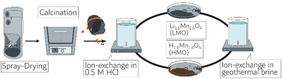

## 1 Introduction

In recent years, there has been a significant rise in the demand for lithium, mainly driven by the automotive industry. This development is likely to continue, causing an increasing gap between lithium demand and supply. The findings of the model by Han et al.^[^ ^1^ ^]^ illustrate that, in a basic scenario, the anticipated global supply of lithium is expected to reach 200 000 tons by 2030. Alternatively, under a high supply scenario, these estimates escalate to 350 000 tons. It is forecast that in Europe alone over 20 battery manufacturing facilities (gigafactories) will be operational by 2040, with an estimated annual production capacity of 600 GWh.^[^ ^2^ ^]^ Currently, lithium is mainly extracted from hard rock deposits and salt lakes (continental brines), with the main production sites and resources located in Bolivia, Chile, Argentina, Australia, and China.^[^ ^3^ ^]^ In 2021, global production reached 100 000 tons, with the South American lithium triangle and Australia contributing significantly.^[^ ^3^ ^]^ To meet the future raw material demand of European gigafactories and to facilitate logistics, mining of pan‐European lithium resources would reduce Europe's resource dependencies and establish domestic value chains. A promising source is geothermal brine because the water is highly mineralized and in some regions enriched with lithium ions. One such region in Germany is the Upper Rhine Graben with particularly lithium‐rich brines with Li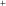 concentrations of up to 210 mg L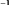.^[^ ^4^ ^]^ Kölbel et al. report that at a production rate of 50 L s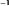 of geothermal brine with a recovery rate of 90% can result in a production of ≈1300 tons of lithium carbonate (Li
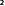
CO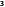) per year.^[^ ^5^ ^]^ In addition to lithium, these brines also contain significant amounts of other ions such as sodium, potassium, and calcium, which makes selective extraction of lithium a challenge. Therefore, development of highly Li
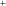
‐selective technologies plays a vital role in overcoming and enabling industrial‐scale extraction of lithium from geothermal brines in the future.

Several direct lithium extraction (DLE) technologies have been considered, including liquid–liquid extraction,^[^ ^6^ ^]^ direct precipitation,^[^ ^7^ ^]^ membrane processes,^[^ ^8^ ^]^ and ion exchange.^[^ ^9^ ^]^ Among these methods, ion‐exchange materials have gained considerable attention as promising candidates for extracting lithium from geothermal brines, since it offers several advantageous features, including high selectivity and a comparatively low energy demand.^[^ ^10^ ^]^ Mn‐based sorbents such as Li
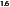
Mn
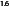
O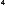 and Li
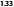
Mn
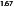
O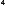 (referred to as lithium manganese oxides [LMOs]) show particularly high selectivities and sorption capacities. However, LMOs exhibit low cycle stability, owing to Mn dissolution and associated degradation of the crystal structure.^[^ ^11^ ^]^ Various routes for the production of LMO with high sorption capacities are described in the literature: Chitrakar et al. synthesized LiMnO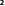 precursor hydrothermally by autoclaving *γ*‐MnOOH with 4 M LiOH at 120 °C for 1 day followed by calcination at different temperatures.^[^ ^9^ ^]^ The material reached sorption capacities of up to 40 mg g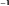. In another study, the LiMnO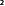 precursor has been synthesized by boiling *γ*‐MnOOH with 4 M LiOH for 8 h under reflux.^[^ ^12^ ^]^ Subsequent calcination at 400 °C yielded LMO with an sorption capacity of 34 mg g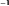. Xiao et al.^[^ ^13^ ^]^ combined a hydrothermal and solid‐state synthesis, to produce Li
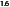
Mn
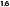
O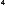 with a high sorption capacity of 6.06 mmol g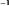 (42.06 mg g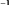) at pH = 10. It is important to highlight that the extraction yield is strongly pH dependent and highest under alkaline conditions. However, this is usually not the case for geothermal brines and hence the resulting sorption capacities are typically below 4 mmol g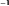. A major bottleneck in the aforementioned approaches is their poor scalability, particularly approaches involving hydrothermal synthesis steps. Therefore, we herein presented an alternative synthesis route, utilizing a spray‐drying process to overcome the scalability constraints faced by existing synthesis methods. This novel approach enables the upscaling of sorbent synthesis and facilitates industrial application, owing to a fast reaction time, high throughput, and the capability for continuous operation. As will be demonstrated herein, this synthesis approach is also well suited to incorporate substituent transition metals into the process to flexibly tailor the material properties. Previous studies introduced a variety of transition metals with similar atomic radii to that of manganese, including elements such as iron,^[^ ^14^, ^15^ ^]^ aluminum,^[^ ^16^ ^]^ cobalt,^[^ ^14^, ^17^ ^]^ nickel,^[^ ^14^ ^]^ and a combination of iron and aluminum.^[^ ^18^ ^]^ The substitution of Mn(IV) by Ti(IV) has shown significant improvements in related battery materials with respect to the long‐term stability of Mn‐based cathodes.^[^ ^19^ ^]^ The benefits of this approach for ion‐exchange materials are presented herein by substituting <5 mol% of Mn by Ti, which improved both the lithium sorption behavior and chemical stability in the extraction process.

## 2 Experimental Section

### 2.1 Preparation of the Material

**Figure 1:**
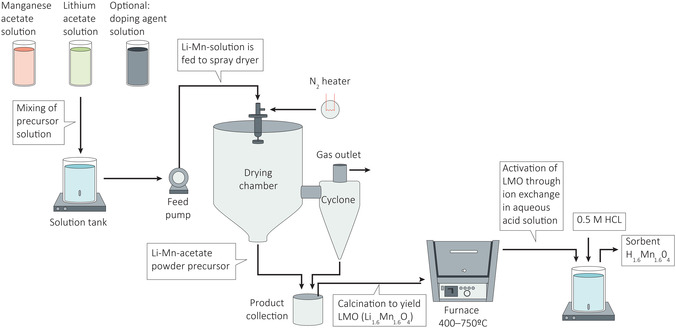
Schematic diagram of material preparation (H
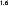
Mn
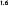
O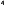) including preparation of Li–Mn solution, spray‐drying process, calcination, and pickling of precursor (Li
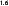
Mn
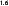
O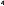) with 0.5 M HCl.

Sorbent material was synthesized via novel spray‐drying process. A schematic illustration of the synthesis pathway is given in **Figure** **1**. The LMO sorbent was prepared according to the following procedure: Lithium acetate solution was prepared mixing 109.17 g (1.07 mol) lithium acetate dihydrate (Li(CH
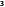
COO) $\cdot 2H_{2}O$, purity: 98%, Acros Organics) with 0.4 L deionized water. Manganese acetate solution was prepared by adding 262.25 g (1.07 mol) manganese(II) acetate tetrahydrate (Mn(CH
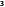
COO)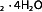, purity: >99%, Sigma Aldrich) in 0.8 L of deionized water. The solutions were mixed and stirred. The mixture was then dried in a spray‐dryer (GEA Niro MOBIL MINOR) using nitrogen as carrier gas, and the inlet and outlet temperatures of the gas were *T* 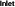 = 210 °C and *T* 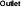 = 112 °C. A coarse fraction of 75.8 g and a fine fraction of 155.5 g were obtained from the spray‐dryer. The powder was mixed again for further synthesis. Subsequently the Li–Mn salt was calcined at different temperatures (800, 720, and 450 °C) for 5 h in air to obtain the lithium ion‐sieve precursor Li
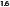
Mn
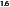
O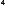. Also stepwise calcination at 720 and 450 °C for each 5 h was performed. The sorbent H
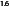
Mn
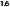
O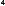 was obtained by pickling Li
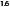
Mn
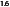
O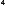 with 0.5 M HCl for 24 h. The suspension was filtered, washed with bidistilled water, and dried for 24 h at 60 °C.

The Ti‐substituted LMO sorbent material (LMTO) was synthesized using a similar route. A lithium acetate solution was prepared by combining 99.58 g (0.976 mol) lithium acetate dihydrate (Li(CH
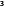
COO) $\cdot 2H_{2}O$, purity: 98%, Acros Organics) with 0.2 L of deionized water. Similarly, a manganese acetate solution was created by dissolving 231.73 g (0.945 mol) manganese(II) acetate tetrahydrate (Mn(CH
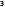
COO)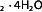, purity: >99%, Sigma Aldrich) in 0.8 L of deionized water. Additionally, 9.09 g (0.032 mol) titanium(IV)‐isopropoxide (Ti[OCH(CH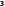)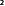]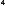, purity: 97%, Aldrich) was dissolved it 0.2 L (3.49 mol) acetic acid (CH
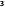
COOH, purity: 100%, Supelco). The solutions were then combined and stirred. The resulting mixture was dried in a spray‐dryer using nitrogen as the carrier gas, with inlet and outlet gas temperatures set to *T* 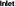 = 210 °C and *T* 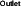 = 112 °C, respectively. From the spray‐dryer, a coarse fraction weighing 36.8 g and a fine fraction weighing 160.6 g were obtained. The powder was subsequently remixed for further synthesis. Next, the Li–Mn–Ti salt was calcined at 450 °C for 5 h in air to produce the lithium ion‐sieve precursor Li
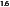
Ti
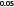
Mn
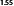
O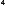. The sorbent H
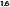
Ti
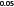
Mn
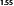
O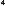 was then obtained by treating Li
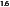
Ti
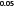
Mn
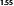
O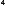 with 0.5 M HCl for 24 h. After filtration, the suspension was washed with bidistilled water and dried for 24 h at 60 °C.

### 2.2 Physical Analysis

X‐ray powder diffraction (XRD) was performed to conduct phase analysis of the sorbent materials. The analysis was performed on a STOE Stadi P powder diffractometer in transmission geometry, using monochromatic Mo‐K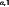 radiation (*λ* = 0.7093 Å) with position‐sensitive detector step of 0.06 and a scanning speed of 40 s step^−1^ in a range from 10° to 72°. Scanning electron microscopy (SEM) was conducted to visualize the surface morphology of the raw and treated sorbent material using a thermal field‐emission SEM (MERLIN, Zeiss) with X‐ray energy‐dispersive spectroscopy capability (EDX, Quantax 400 SDD, Bruker). Particle surface areas were determined by nitrogen physisorption, using a surface analyzer (Gemini VII 2390a, Micromeritics). The total specific surface area (SSA) was determined following the multipoint Brunauer Emmer‐Teller (BET) method. Simultaneous thermal analysis (thermogravimetric analysis [TGA]‐differential thermal analysis (DTA) 200 mg/differential scanning calorimetry [DSC]) with combined Fourier transform infrared gas analysis operated under Ar atmosphere (Netzsch Jupiter 449 C) was used for bulk characterization.

### 2.3 Chemical Analysis

The ion concentrations of Li, Mn, and competing ions were analyzed by inductively coupled plasma optical emission spectroscopy (ICP–OES, 700 Series‐Agilent Technologies). For analyzing the material composition, the sorbent was dissolved by mixing the powder with 37 wt% HCl (Merck KGaA) and 30% H
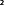
O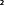 (Carl Roth GmbH). To prepare the samples for ICP–OES measurement, 1 mL of sample was added to 0.1 mL 65% nitric acid (HNO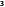) and 9 mL of distilled water. The calibration of the measuring instrument was conducted using multielement standards at different concentrations (1, 10, 25 mg L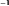) provided by Merck. Additionally, the ion concentrations of a blank and the quality standard were measured. If the concentration of a sample exceeded the calibrated measurement range, it was further diluted with distilled water.

The standard deviation in the cation concentrations obtained from ICP–OES measurements yielded both 0.8% for lithium and manganese, respectively. Three different samples of the LMO‐450 material were characterized in separate sample preparation steps (starting from dissolution of the solid powder, see previous section). The resulting analyte solutions were characterized by ICP–OES in triplicate.

### 2.4 Geothermal Brine

The geothermal brines used in this study originate from the geothermal plant in Bruchsal, Germany (EnBW‐Energie Baden‐Württemberg) and the geothermal plant in Soultz, France (Électricité de Strasbourg, ÉS). Both brines were characterized by a high salt content of >100 g L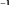 total dissolved solids (TDSs) and a high gas content.^[^ ^4^ ^]^ The components of the geothermal brines from Bruchsal and Soultz, based on literature data, are provided in **Table** **1**. Geothermal brine, with its high salt content, remained stable only under stringent conditions, such as those prevailing in geothermal systems: high pressure, elevated temperature, and the exclusion of oxygen. Experiments in this work were performed at room temperature. Due to temperature change and prolonged storage of the geothermal brines over several months, their composition evolved compared to the values reported in the literature. The precise composition of the water used in these experiments is presented in Table S3, Supporting Information.

**Table 1:** Composition of the geothermal brine Bruchsal (EnBW)^[^ ^5^ ^]^ and Soultz (ÉS)^[^ ^36^ ^]^ based on literature data.

Location	c	Li	Na	K	Mg	Ca	Sr	Ba	Pb
Bruchsal	[mg L 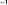 ]	155	36 000	3200	320	6900	350	5.9	3.2
Soultz	[mg L 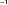 ]	163	26 700	3350	123	7030	434	22	0.16

### 2.5 Li‐Sorption Experiments

Various lithium extraction tests were conducted using H
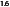
Mn
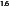
O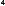 (referred to as HMO) and Ti‐substituted (HMTO) sorbents. The materials were exposed to lithium‐containing solutions, comprising synthetically prepared LiCl solution (c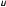 = 143 mg L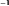) and geothermal brine from the Bruchsal geothermal plant. Initial assessments with HMO calcined at different temperatures were performed to evaluate the influence of calcination temperature on lithium extraction capabilities. Subsequent investigations were carried out with HMO and HMTO calcined at the optimal temperature of 450 °C. The experiments included the pH dependence on the extraction process, the impact of the mass‐to‐volume ratio (m/V ratio) on the extraction capacity, as well as the impact of the initial lithium concentration in the brine. In addition, the study examined the ion‐exchange kinetics, long‐term performance in terms of sorption/desorption capacities and Mn dissolution, and Li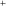 selectivity of the ion‐exchange process. A comprehensive overview of the different experiments is provided in **Table** **2**.

**Table 2:** Overview of the various ion exchange experiments: type of experiments, amount of used sorbent mass, concentration (c), and volume of brine. Solutions with buffering agent are indicated by a (b).

Experiment	Mass sorbent	Li 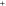 solution	c 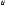 [mg L 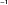 ]	Volume
Initial sorption capacity	200 mg	LiCl solution (b)	143	40 mL
pH dependence	200 mg	LiCl solution	143	40 mL
	200 mg	Bruchsal brine	143	40 mL
	200 mg	Bruchsal brine (b)	142	40 mL
m/V ratio	Variation	LiCl solution (b)	143	40 mL
Variation of c 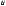	200 mg	LiCl solution (b)	46, 63, 142, 197	40 mL
	200 mg	Bruchsal Brine (b)	143	40 mL
	200 mg	Soultz Brine (b)	142	40 mL
Kinetics	200 mg	LiCl solution (b)	143	40 mL
Ion‐exchange selectivity	200 mg	Bruchsal Brine (b)	143	40 mL
Cycling 1	200 mg	LiCl solution (b)	143	40 mL
Cycling 2	200 mg	Bruchsal Brine (b)	143	40 mL

#### 2.5.1 Initial Sorption Capacity

To investigate the impact of calcination temperature on Li
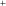
‐sorption performance, sorption tests were conducted. A LiCl–NaOAc stock solution was prepared with a Li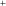 concentration of 143 mg L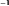, and 1.5 g L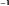 (18.3 mmol L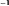) sodium acetate (NaOAc) was included as a buffering agent. For each test, 200 mg of HMO sorbent, calcined at different temperatures (450 °C; 720 °C; stepwise 720 and 450 °C; and 800 °C), was added to 40 mL of the LiCl solution and stirred for 24 h. Following the Li
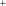
‐sorption step, the solid was separated via centrifugation, and ion concentrations in the supernatant were analyzed with ICP–OES. In addition, the same experiment was carried out with HMTO calcined at 450 °C.

#### 2.5.2 pH‐Dependence of Ion Exchange

For pH‐dependence experiments LiCl solution was prepared with Li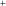 concentration of 143 mg L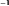. Different pH values between 1 and 12 were adjusted by adding NaOH or HCl. An amount of 200 mg of sorbent was mixed with 40 mL of LiCl solution and stirred for 24 h. Subsequently, the solid was separated by centrifugation and the ion concentrations in the supernatant were analyzed using ICP–OES. Furthermore, following the same procedure, an experiment was conducted using both unbuffered geothermal brine and buffered geothermal brine (with NaOAc concentration of 1.5 g L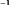).

#### 2.5.3 Sorbent‐to‐Brine Ratio (m/V Ratio)

The correlation between the ratio of mass of sorbent to volume of LiCl solution with respect to sorption capacity and lithium extraction efficiency was investigated. Varying amounts of the sorbent material (10, 100, 400, and 1000 mg) were suspended in a beaker with 40 mL of a LiCl–NaOAc solution (c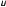 = 143 mg L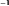) each. The NaOAc concentrations of the LiCl–NaOAc solution was either c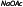 = 1.5 g L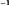 (18.3 mmol L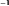) and c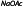 = 15 g L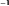 (183.8 mmol L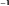). The suspension was stirred for 24 h, before the solid fraction was separated by centrifugation. Then Li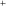 concentration in the supernatant was determined by ICP–OES for the calculation of the sorption capacity.

#### 2.5.4 Li Extraction Using Various Li Concentrations and Geothermal Brine

The influence of the initial lithium concentration of the starting solution on the sorption capacity was determined. Various LiCl solutions with different Li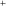 concentrations (46, 63, 142, and 197 mg L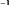) were prepared. Experiments with natural brines from the geothermal plants in Bruchsal (c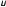 = 143 mg L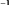, EnBW) and Soultz (c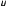 = 141 mg L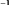, ÉS) were carried out to assess the ion‐exchange process under conditions of high salinity and interfering cations. For each experiment 200 mg HMO was suspended in 40 mL of brine and stirred for 24 h. Then the solid material was isolated through centrifugation, and the ion concentrations in the resulting supernatant were analyzed using ICP–OES.

#### 2.5.5 Ion‐Exchange Kinetics

Kinetic experiments were carried out by suspending 200 mg HMO or HMTO with 40 mL of buffered synthetic brine (c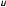 = 143 mg L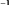, c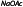 = 1.5 g L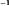). The reaction time (5, 10, 15, 20, and 30 min and 1, 2, 4, and 24 h) was varied to determine the ion‐exchange rate from the concentration of Li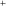 ions in the brine by ICP–OES. After the designated exposure times, solid and liquid phases were separated via centrifugation, and the supernatant was analyzed using ICP–OES.

To describe the reaction kinetics, the pseudo‐first‐order and pseudo‐second‐order kinetic models, as presented Equation (1) and (2), were employed and correlated with the results obtained from the kinetic experiments.

(1) $$\ln\left(q_{e}-q_{t}\right)=\ln\left(q_{e}\right)-k_{1}\cdot t+\ln\left(q_{e}\right)$$

(2) $$\frac{t}{q_{t}}=\frac{1}{k_{2}\cdot {\left(q_{e}\right)}^{2}}+\frac{t}{q_{e}}$$

The sorption capacity after reaction time *t* is indicated with *q* 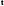. The equilibrium‐sorption capacity is signified with *q* 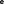, the reaction constants with *k* 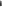.

The data fitting was performed with the Python library SciPy. The algorithm was optimized using the nonlinear least squares method (Figure S6, Supporting Information).

#### 2.5.6 Cycling Tests/Ion‐Exchange Selectivity

Cycling tests were conducted by adding 200 mg HMO or HMTO to 40 mL of buffered LiCl solution (c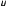 = 143 mg L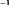, c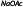 = 1.5 g L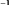) or buffered geothermal brine (c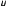 = 143 mg L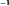, c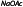 = 1.5 g L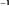). After stirring for 30 min, the solution was separated from the solid material via centrifugation and a sample of the supernatant was taken for ICP–OES measurements. The solid fraction was washed with distilled water two times. Subsequently, desorption was carried out by addition of 40 mL 0.5 M hydrochloric acid (HCl) solution. The suspension was stirred for 30 min at room temperature and then separated and washed as described earlier. The supernatant was analyzed for Li, Mn, and competing ions using ICP–OES. All calculations of sorption capacity are based on the sorbent mass determined prior first cycling experiment. Sorption and desorption were repeated for 5 cycles. The material was then analyzed using X‐ray diffraction (XRD) and SEM to assess the impact of cyclic usage on the crystal structure and morphology.

Selectivity analyses were conducted with the solutions obtained from the first cycle of the long‐term experiments.

## 3 Calculations

### 3.1 Ion‐Exchange Capacity *Q* Sorb

The lithium content in the sorbent materials, capacity *Q* 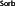, was calculated according to Equation (3).

(3) $$Q_{\text{Sorb}}\left[\frac{\text{mmol}}{g}\right]=\left(c_{0}-c\right)\cdot \frac{V}{m_{s}\cdot M_{\text{Li}}}$$

where *V* is the volume of the solution, *m* 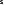 is the initial mass of the sorbent, *M* 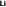 is the molar mass of Li, *c* 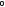 is the initial concentration of Li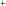, and *c* is the concentration after sorption.

### 3.2 Li‐Depletion Factor, *R*_D_

The lithium‐depletion factor *R* 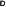 describes the extent to which the initial lithium content in the brine can be captured by the material. It was calculated based on the difference in Li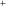 concentration before (*c* 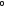) and after sorption (*c*), relative to the initial Li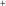 concentration (*c* 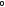) as indicated in Equation (4).

(4) $$R_{D}\left[\%\right]=\frac{\left(c_{0}-c\right)}{c_{0}}$$

### 3.3 Transfer Factor *T*_CI_

The molar fraction of competing ions (geothermal brines only) that end up in the desorption solution is given by the transfer factor $T_{\text{CI}}$. It can be determined from the ratio of the concentrations of any competing ion (e.g., Na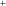) in the desorption solution and original geothermal brine, according to Equation (5).

(5) $$T_{\text{CI}}=\frac{n_{\text{Me,De}}}{n_{\text{Me,0}}}$$

The amount of alkali and alkaline earth metals in the desorption solution are represented by *n* 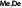 and the initial amount of metal ions in the geothermal brine by $n_{\text{Me,0}}$.

### 3.4 Mn Dissolution and Ti Dissolution

The manganese dissolution can be calculated from the ratio of the concentration of dissolute Mn(II) *c* 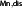 to the volume of the desorption solution *V* 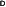 and the total amount of Mn in the sorbent *n* 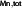 according to Equation (6). The calculation for Ti is analogous.

(6) $$D_{\text{Mn}}\left[\%\right]=\frac{c_{\text{Mn,dis}}\cdot V_{D}}{n_{\text{Mn,tot}}}\cdot 100=\frac{n_{\text{Mn,dis}}}{n_{\text{Mn,tot}}}\cdot 100$$

## 4 Results and Discussion

### 4.1 Material Synthesis and Characterization

**Figure 2:**
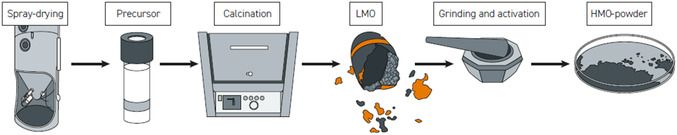
Synthesis of the sorbent material: after a spray‐drying process, a Li–Mn–O precursor is obtained and calcined to receive LMO pristine material. After grinding and activation, the ready HMO sorbent is obtained.

The LMO sorbent with the targeted composition of Li
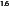
Mn
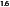
O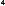 was synthesized in a two‐step process as described earlier and schematically shown in **Figure** **2**. In brief, a precursor was prepared by spray‐drying from a solution containing Mn(II) acetate and Li acetate and then calcined. TGA of the precursor was performed to select appropriate temperatures for the calcination step. The precursor was then calcined at the selected temperatures and subsequently analyzed by XRD and SEM. The materials were activated, and tested for their sorption capabilities.

#### 4.1.1 TGA and DSC

**Figure 3:**
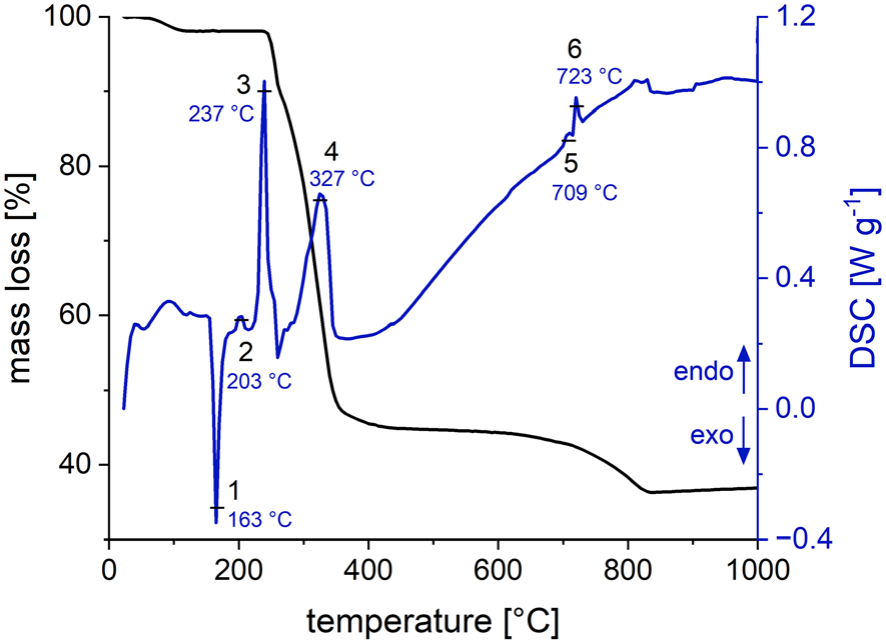
Analysis of mass loss and heat flow during heating of LMO precursor in a TGA.

The thermograms from TGA and DSC analysis of the precursor (**Figure** **3**) show a multistage decomposition process starting with minor water evaporation at about 100 °C (≈2% mass loss). The DSC heat flow further indicates an exothermic process at 163 °C and two endothermic processes at 202 and 237 °C without any changes in mass. A first decomposition is observed at temperatures exceeding 250 °C, up until ≈420 °C, and is accompanied by a substantial mass loss of ≈55%. The DSC signal (4) exhibits tailing toward lower temperatures, suggesting that the degradation proceeds faster as the temperature increases. During this phase, the organic components of the precursor decompose and the oxide phase forms, which shows minimal mass change up to 700 °C. Two phase transitions then occur at 708 and 722 °C respectively, followed by a further decomposition reaction resulting in an ≈8% mass reduction.

The results are in good agreement with earlier studies reporting optimal calcination temperatures around 450 °C in hydrothermal synthesis approaches.^[^ ^20^ ^]^ Herein, calcination temperatures of 450, 720, and 800 °C were initially chosen for the second step of the sorbent synthesis. In addition, a stepwise calcination at 450 and 720 °C was performed. The calcination duration was 5 h as reported previously.^[^ ^20^ ^]^

#### 4.1.2 Chemical Composition and Stoichiometry

The stoichiometry of the resulting LMO compounds under different calcination conditions was analyzed by ICP–OES measurements to determine the Li/Mn ratio of the material (**Table** **3**). At the lowest calcination temperature (450 °C), the Li/Mn ratio was slightly higher (1.02) than for higher calcination conditions (0.98).

**Table 3:** Synthesis parameter of the sorbent material including calcination time and temperature; experiments were conducted using a buffered LiCl solution (c = 143 mg L, c = 1.5 g L, pH = 7.4).

Sample	*T* 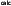 [°C]	*t* [h]	LP LM(T)O [Å]	LP HM(T)O [Å]	Calc. molecular formula ‐	*Q* 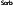 [mmol g 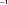 ]	*Q* 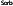 [mg g 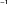 ]	BET [m 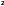 g 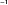 ]^a^^)^
LMO‐800	800	5	8.224	8.071	Li 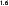 Mn 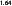 O 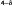	0.39	2.71	1.40
LMO‐720	720	5	8.175	8.072	Li 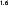 Mn 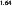 O 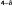	1.21	8.40	2.90
LMO‐720‐450	720, 450	5, 5	8.177	8.067	Li 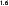 Mn 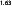 O 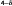	1.63	11.31	2.26
LMO‐450	450	5	8.132	8.061	Li 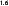 Mn 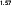 O 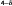	2.03	14.10	2.60
LMTO‐450	450	5	8.142	8.056	Li 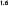 Mn 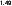 Ti 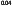 O 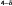	2.22	15.41	6.36

a) Characterization of the sorbents: lattice parameter (LP) of LM(T)O and HM(T)O, the calculated molecular formula (error Li: 0.8%; Mn: 0.8%), sorption capacity *Q* 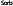, and BET surface area.

#### 4.1.3 Structural Analysis by XRD

**Figure 4:**
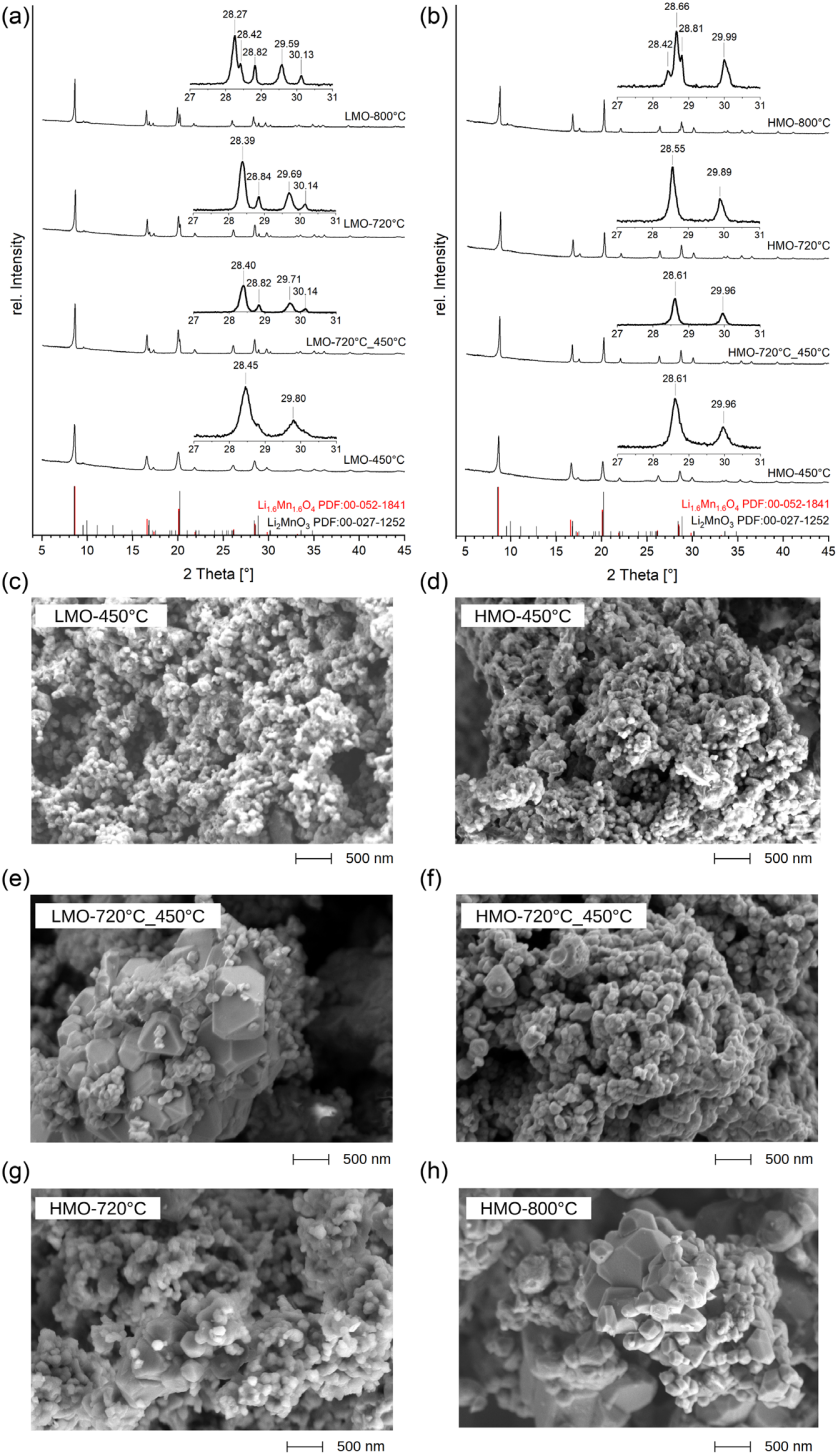
a) X‐ray diffraction patterns of LMO and b) HCl‐treated HMO synthesized at different calcination temperatures. c) SEM of LMO precursor prepared at calcination temperature of 450 °C. d) SEM of HMO calcined at 450 °C after pickling. e) SEM of LMO precursor prepared at step‐wise calcination at 720 followed by 450 °C. f) SEM of HMO prepared at step‐wise calcination at 720 and 450 °C after pickling. g) SEM of HMO calcined at 720 °C after pickling. h) SEM of HMO calcined at 800 °C after pickling.

**Figure** **4**a shows the XRD patterns of the pristine LMO after calcination at different temperatures. The diffraction patterns of the samples calcined at 450 °C match well with the cubic spinel structure of the Li
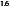
Mn
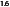
O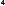 reference pattern (ICDD 00‐052‐1841) with the Fd$\bar{3}$m space group. All characteristic reflections of the spinel are present. A lattice parameter of 8.132 Å was determined which is slightly smaller than the value specified in literature (8.14 Å).^[^ ^20^, ^21^ ^]^ This value is more indicative of an LMO with lower lithium content, such as Li
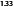
Mn
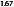
O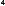.^[^ ^22^ ^]^ At higher‐temperature additional reflections are observed and their intensity becomes more significant as the calcination temperature increases. This can be attributed to the fact that in addition to the spinel phase, a layered oxide phase with distinct monoclinic superstructure reflexes is formed at temperatures above 450 °C. The layered oxide phase can be approximated by the composition Li
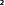
MnO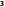, which is a particularly Li‐rich phase. In the spinel phase, an increasing lattice parameter can be observed with increasing calcination temperature (Table 3), reaching 8.224 Å at 800 °C, which is ascribed to a decreasing Li occupancy in the Mn site, as previously described in literature.^[^ ^23^, ^24^, ^25^ ^]^ As higher calcination temperatures result in the formation of larger fractions of the Li‐rich‐layered oxide, the targeted spinel phase is slowly depleted of lithium.

For structural analysis Rietveld refinement was performed on the diffraction data of the LMO‐450, assuming a single‐phase material with a spinel structure (Figure S5a, Supporting Information). For LMO‐800, the refinement was performed using a mixed phase comprising a spinel and a layered structure (Figure S5a, Supporting Information). For the spinel phase, the initial structural model ICDD 1514017 with space group Fd$\bar{3}$m was considered. For layered structured impurity‐phase Li
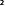
MnO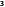 with space group C2/m, the structural model ICDD 1514044 was used. The final refinement yielded $\chi^{2}$ = 3.2 for LMO‐450 and $\chi^{2}$ = 2.2 for LMO‐800, with good agreement of the crystallographic data. A more detailed analysis of the complex, mixed structure will be the subject of future work.

The thus prepared LMO samples were then activated by dispersing the powders in a 0.5 M HCl solution (pickling). Pickling leads to ion exchange of Li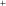 and H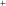 according to Equation (7), yielding the protonated ion‐exchange material HMO. The corresponding XRD patterns are shown in Figure 4b. In general, pickling leads to a shift of the reflections to wider angles for all samples and thus a reduction of the lattice parameter. For material calcined at 450 °C, lattice parameter is reduced to 8.061 Å which matches well with literature.^[^ ^20^ ^]^ Interestingly, the Li‐free HMOs show only spinel phase but no layered phase for HMO‐720 and HMO‐720‐450, suggesting that the HCl‐treatment selectively dissolved the Li
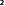
MnO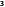 phase impurity. HMO‐800 is the only material that shows residual phase impurities after pickling.

(7) $${\text{Li}}_{1.6}{\text{Mn}}_{1.6}O_{4}+1.6\;H^{+}⇌H_{1.6}{\text{Mn}}_{1.6}O_{4}+1.6\;{\text{Li}}^{+}$$

#### 4.1.4 Particle Characterization

Figure 4c presents the SEM image of LMO calcined at 450 °C. The morphology of HCl‐treated sorbent HMO is shown in Figure 4d. For most particles, the particle size for LMO and HMO calcined at 450 °C is smaller than 150 nm (Figure S9, Supporting Information). The treatment of sorbent with 0.5 M HCl does not affect the particle size. Figure 4e,f compare the morphologies of LMO and HMO material that were calcined stepwise at 720 and 450 °C. The micrographs of the LMO‐750‐450 sample display spherical particles with sizes <150 nm that are distributed on larger octahedral crystals of up to 1 μm in size. As already confirmed by XRD analysis, a second‐layered Li
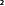
MnO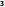 phase is formed at higher temperatures that can be ascribed to the second type of particles seen in the micrographs. After pickling, only a few of these large crystals remain visible suggesting that the Li
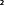
MnO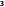 phase is dissolved in the pickling process. Lastly, Figure 4g,h show SEM images of HMO powder after pickling of LMO‐720 and LMO‐800. Although HMO‐720 (Figure 4g) shows a similar morphology than the HMO‐720‐450, few minor Li
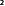
MnO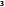 residuals are visible as larger crystalline particles. It is thus consistent, that HMO‐800 powder (Figure 4h) exhibits the largest fraction of the residual Li
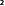
MnO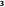 phase, despite the treatment in 0.5 M HCl.

For all materials an increasing particle size with increasing calcination temperature can be seen. Comparable results were observed from XRD measurements regarding the crystallite sizes. The broadening of the diffraction peaks at lower calcination temperatures suggests the presence of smaller crystallite sizes. The corresponding BET SSAs correlate with these findings, since with decreasing calcination temperature, the particle size decreased and accordingly the SSA increased. The LMO sample calcined at 450 °C shows SSA of 2.60 m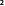 g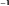, whereas at 800 °C the SSA decreased to 1.40 m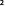 g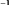. The BET SSA are summarized in Table 3. Details of the BET analysis parameters are provided in Table S1, Supporting Information, while the corresponding isotherm linear plots and BET surface area curves are shown in Figure S1 and S2, Supporting Information, respectively.

#### 4.1.5 Ion‐Exchange Capabilities

The calcined LMO samples were investigated for their lithium sorption capabilities. After activation to the corresponding HMOs, they were suspended in a synthetic LiCl solution (c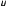 = 142 mg g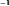). The ion‐exchange capacity was determined by measuring the change in Li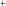 concentration between the original LiCl solution and in the supernatant after separation of the sorbent. In this and most of the following experiments, a buffer agent (Na acetate, NaOAc) was added to the LiCl solution that forms an acetate buffer in situ during the ion exchange with HMO. This was deemed necessary, as pH of the Li
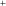
‐rich solution would drift rapidly to low pH and thus ceasing the ion‐exchange process. The acetate buffer maintained a pH that is characteristic for the geothermal brines in Table 1. More details on the pH dependency of the ion‐exchange process are provided in the sections later. The sorption capacities in this experiment are provided in Table 3 and were highest for LMO‐450 (14.1 mg g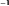). The capacities decreased from the lowest to the highest calcination temperature and were particularly low for LMO‐800 with *Q* 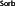 = 2.71 mg g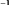. The materials LMO‐450, LMO‐720, and LMO‐720‐450 maintain a nearly constant SSA, while sorption capacity significantly decreases. This suggests that particle size has a limited effect on lithium uptake. As outlined earlier, the poorer sorption capacity is likely a result of the prominent Li
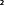
MnO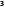 phase in this material. Li
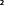
MnO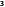 does not seem to act as an effective ion‐exchange material and would fully dissolve upon repeated extraction experiments.

#### 4.1.6 Ti Substitution

In an effort to improve the chemical stability of the spray‐dried LMO‐450, i.e., to reduce Mn dissolution, a substitution approach was tested. Gao et al. have demonstrated that in addition to Mn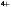 a small amount of Mn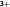 is present in Li
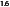
Mn
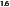
O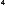, which causes the Mn dissolution.^[^ ^11^ ^]^ Disproportionation effects (2Mn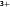 → Mn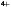 + Mn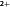) result in a dissolution of water‐soluble Mn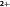, leading to irreversible loss of sorbent. Conversely, the presence of Mn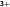 in LMO is accompanied by the Jahn–Teller effect, which impacts the stability of the structure.^[^ ^26^ ^]^ When substituting a small fraction of Mn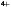 in the material, these two adverse effects can be largely suppressed, thus contributing to improved structural stability. Herein, Ti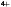 was chosen for substitution because its ion radius (0.605 Å) is close to the Mn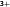 radius (0.645 Å, high spin),^[^ ^27^ ^]^ ensuring compatibility of heteroatoms with the original lattice.^[^ ^28^ ^]^ Additionally, other studies using Fe,^[^ ^15^ ^]^ K,^[^ ^28^ ^]^ and Zn^[^ ^29^ ^]^ have reported to stabilize the host structure in molar amounts as low as a few mol%. Therefore, in this study, a molar Li/Ti ratio of 32 and a Ti/Mn ratio of 0.032 were targeted. Ti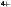 was introduced into the precursor by first preparing a solution of titanium tetraisopropoxide and acetic acid, which was then mixed with the Mn‐ and Li‐acetate solution to prepare the precursor material for the calcination step. This ensured a thorough mixing of the transition metals and a homogeneous “Li‐Mn‐Ti” precursor after spray‐drying. After calcination at 450 °C, a black powder was obtained (labeled LMTO) with a Li/Mn ratio of 1.07, a Li/Ti ratio of 36.2, and a BET SSA of 6.36 m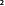 g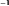. Figure S3a, Supporting Information, shows the diffractograms of the Li–Mn–Ti precursor, the calcined LMTO, and activated HMTO. The precursor is an amorphous powder with broad background. The diffraction patterns of LMTO align with the cubic spinel structure of the Li
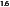
Mn
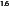
O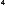 reference pattern (ICDD 00‐052‐1841), exhibiting an Fd$\bar{3}$m space group. All discernible reflection characteristics of the spinel structure are distinctly present. The lattice parameter of LMTO (8.142 Å) was slightly larger than for LMO (with 8.132 Å), which is consistent with a slight shift of the reflections toward smaller angles. The LMTO was then treated with 0.5 M HCl to obtain the activated HMTO for ion‐exchange experiments. The corresponding XRD pattern exhibits a shift of reflections toward wider angles and a decrease in the lattice parameter to 8.056 Å, which is comparable to the Ti
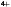
‐free H
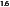
Mn
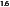
O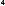 (HMO, 8.061 Å).

The Ti‐substituted sorbent was investigated for its Li
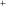
‐sorption capabilities (ion‐exchange capabilities). An sorption capacity of 15.41 mg L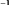 was measured which is higher than the sorption capacities of the Ti
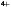
‐free materials (2.71–14.1 mg g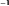, Table 3) indicating an improved ion‐exchange behavior compared to LMO.

Figure S3b, Supporting Information, shows an SEM image of the pristine LMTO. SEM analysis reveals the presence of small particles exhibiting platelet‐like structures, with diameters approximately ranging from 50 to 200 nm. The platelet‐like shapes disappeared after the activation step (Figure S3c, Supporting Information). The resulting HMTO exhibited more uniform and predominantly spherical particles. In addition, a narrower particle size distribution was observed. As evident from complementary EDX mapping (Figure S3d–f, Supporting Information), a homogeneous distribution of Ti (d), O (e), and Mn (f) was achieved by our synthesis approach.

### 4.2 Li‐Extraction Capabilities

In the following, the HMO and HMTO materials calcined at 450 °C were used for further experiments with synthetic LiCl solutions and geothermal brines. Unlike other sources of Li‐rich solutions, the extraction from geothermal brines is performed under less favorable pH conditions, i.e., under mild acidic conditions (pH ≈ 5.5). Their respective pH values and buffer capacity to external changes in proton concentrations can vary significantly as a result of different compositions at different extraction wells. In mild acidic environments, the sorbent is less potent, because the Li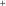/H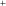 exchange is an equilibrium reaction that strongly depends on concentration gradients between bulk sorbent phases and brine. For example, at high pH, protons are quenched by OH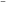 in the exchange process from HMO to LMO, creating a strong thermodynamic driving force. For this reason, most studies report their sorbent capacities at pH values above 10.^[^ ^13^, ^21^ ^]^ Therefore, the impact of pH, (sorbent) mass‐to‐brine ratio (m/V ratio), and initial lithium concentration on the extraction efficiency is investigated below in more detail for a batch extraction process with the aim to find improved conditions for high ion‐exchange kinetics, selectivity, and stabilities over several extraction cycles.

#### 4.2.1 pH Dependence

Titration‐type sorption experiments were conducted to investigate the influence of pH on the Li
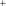
‐sorption capacity of HMO and HMTO.

**Figure 5:**
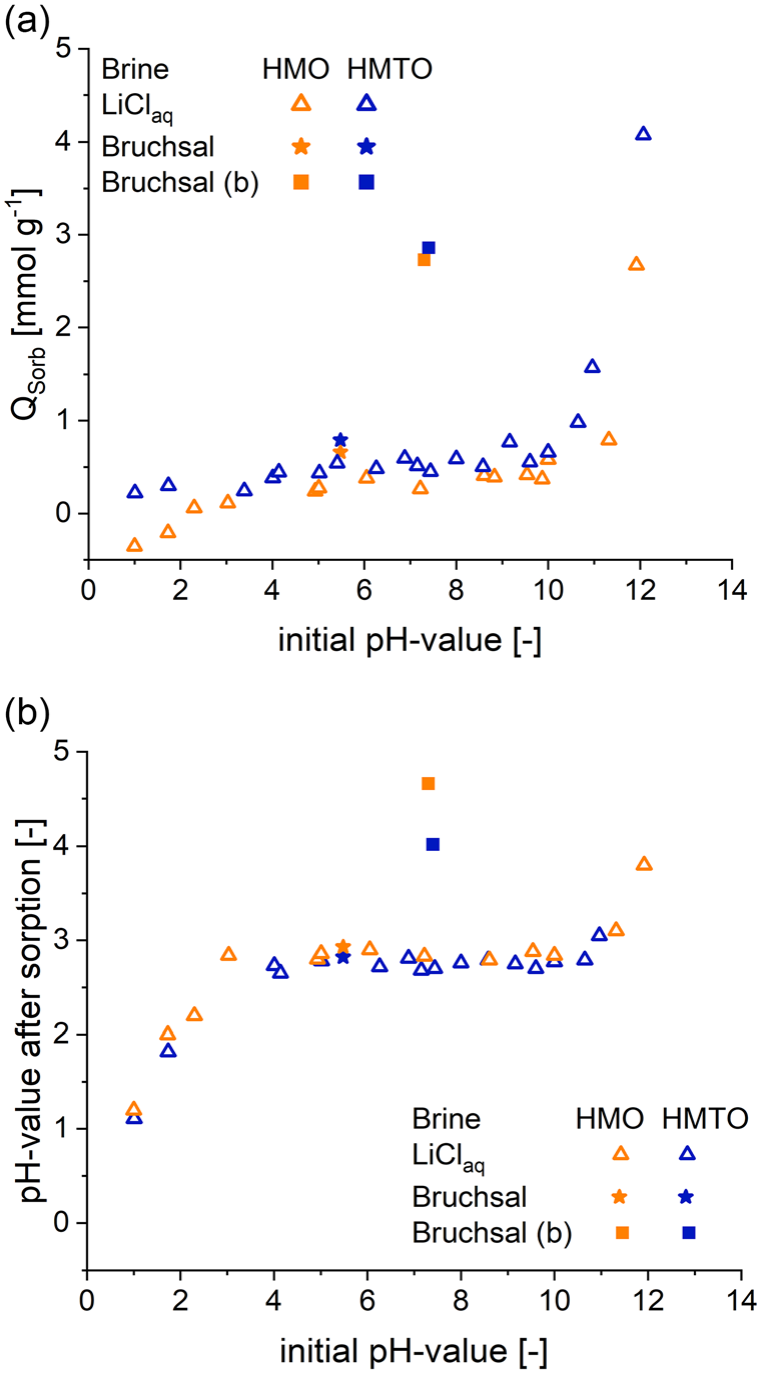
a) The sorption capacity of LMO and LMTO at different initial pH levels is shown and b) the pH value after sorption is presented. HMO data is shown in orange, and HMTO sample data is shown in blue.

A LiCl solution with c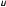 = 143 mg L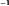 (pH = 7.1) was prepared and the pH was adjusted to various pH levels between 1 and 12 (initial pH values) using HCl or NaOH. HMO or HMTO were exposed to the solutions for 24 h before pH and Li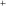 concentration of the solutions were determined. The sorption capacities, *Q* 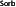, are reported in **Figure** **5**a.

For HMO a negative sorption capacity was observed at a pH below 2, indicating that Li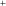 is released from the sorbent. *Q* 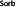 inclines slowly up until a pH value of 10, before a steep change sets in. The highest capacities were measured at a pH of around 12 with a maximum *Q* 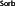 = 2.67 mmol g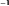 (18.4 mg g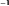) for HMO and a notably larger Li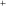 content of *Q* 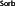 = 4.08 mmol g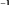 (28.15 mg g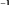) for HMTO. For better comparison, **Table** **4** summarizes *Q* 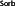 values at different pH for a selected number of ion‐exchange materials with substutuent ion (X), HMXO, reported in literature, including the trivalent ions Al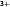,^[^ ^16^ ^]^ Ni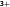,^[^ ^14^ ^]^ Fe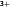,^[^ ^14^ ^]^ and Co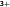.^[^ ^14^ ^]^ At low‐pH (pH = 4) HTMO showed the highest sorption capacity in this comparison (0.45 vs 0.19–0.36 mmol g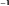), while in the mild basic region other HMXOs displayed higher *Q* 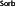 already at lower pH values, as seen from the *Q* 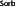 values at pH = 10.

**Table 4:** Comparison of pH dependence on Li‐sorption capacities of HMTO and literature values for selected substituted HMXO (*X* = Al, Fe, Co, Ni) materials.

Substituent X in HMXO	Q_Sorb_ (mmol g^−1^) at different pH				*m*/*V* [g L^−1^]	Source
	pH = 4	pH = 7	pH = 10	pH = 12		
Al	0.19	0.4	1.61	4.24	1	[16]
Fe	0.36	0.55	1.41	3.31	1	[14]
Co	0.3	0.45	1.46	3.96	1	[14]
Ni	0.35	0.59	1.51	3.36	1	[14]
Ti	0.45	0.52	0.82	4.08	5	–

Figure 5a shows a correlation between the sorption capacity and the pH values. Over a broad range of initial pH values, an equilibrium state is observed around the pH of 2.8 (HMO) and 2.7 (HMTO). These results are in agreement with previously reported data by Shi et al.^[^ ^30^ ^]^ who used synthetic LiCl solutions with various starting pH (adjusted by LiOH or HCl) to determine the equilibrium pH. Only as the initial pH value of the LiCl solution (Figure 5b) enters stronger basic environments, the equilibrium pH starts to increase.

In addition to the experiments using LiCl solution, a sorption experiment was conducted with unbuffered geothermal brine (initial pH 5.5) under otherwise identical conditions. The sorption capacity and the pH change after extraction are added in Figure 5 (squared data points). Both the sorption capacity and equilibrium pH were markedly higher with geothermal brine compared to the LiCl solution with similar pH. This can be attributed to the high TDS (>100 g L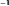) and carbonate content of the geothermal brine forming a buffer system. This effect was observed previously by Zhang et al.^[^ ^31^ ^]^ in salt lake brine as well. The results demonstrate that in batch‐type extractions the pH is drifting significantly without further pH control. In contrast, geothermal brines would suffer from considerable degrees of precipitation if the pH was changed to values above pH > 10. Therefore, an alternative strategy is to buffer the brine during the Li
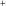
‐sorption process at a pH above the equilibrium pH of 2.7 (2.8), for example with acetic acid as reported previously.^[^ ^32^ ^]^ Herein, sodium acetate (pK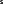 = 4.76) with a concentration of 1.5 g L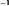 was added to the brine before the sorption tests. The acetate buffer is then generated in situ with the release of protons from the ion‐exchange material. The addition of NaOAc raised the initial pH of the geothermal water from 5.5 to ≈7.4. After the sorption step an equilibrium pH of 4.66 (HMO) and 4.02 (HMTO) was measured, respectively. In addition, the sorption capacities increased notably to 2.7 mmol g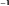 (HMO) and 2.9 mmol g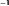 (HMTO) compared to 0.3 mmol g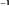 (HMO) and 0.5 mmol g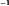 (HMTO) in unbuffered LiCl solutions. For sorption experiments in following sections, the NaOAc buffer agent will be used and further investigated as a means of pH control.

#### 4.2.2 Sorbent‐to‐Brine Ratio (m/V Ratio)

**Figure 6:**
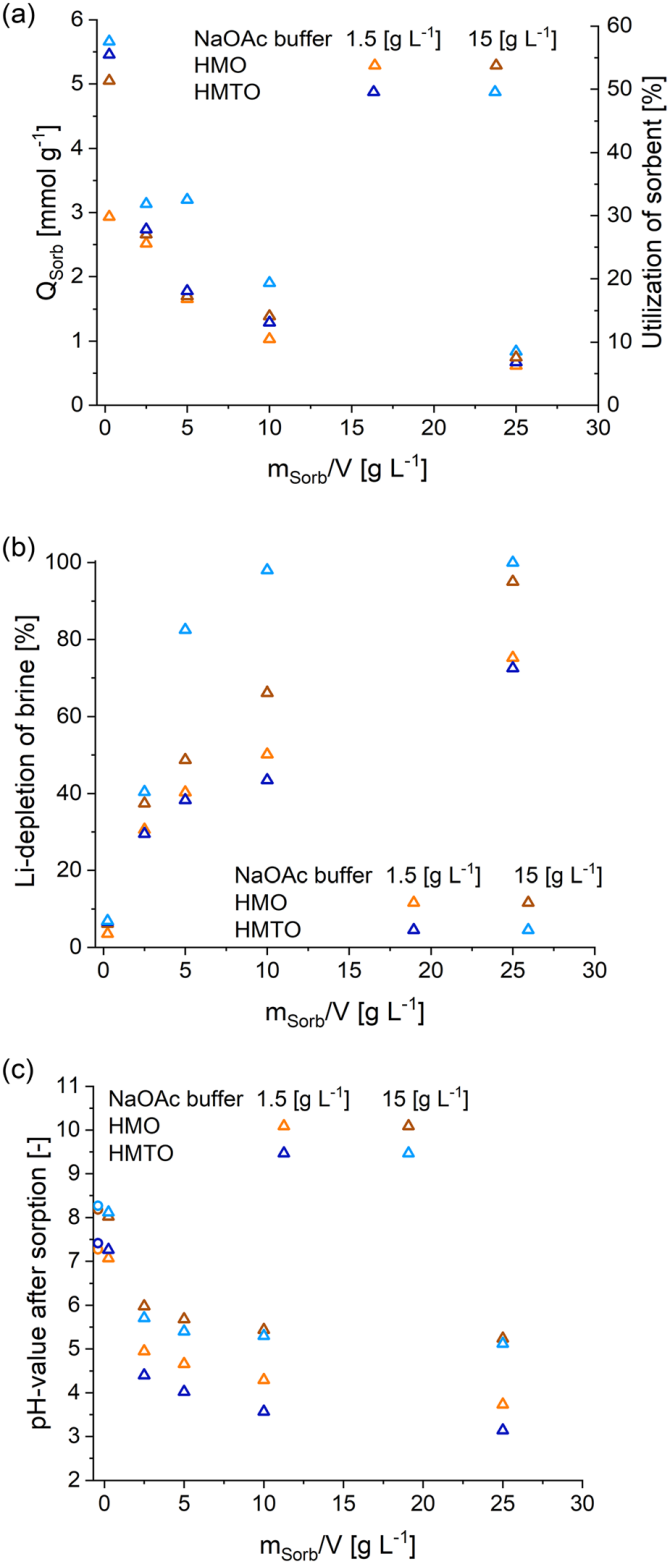
Variation of mass to volume ratio: a) sorption capacities, b) Li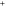 depletion of brine, and c) initial pH values (circle) and pH values after sorption (triangle) are presented. Data for HMO is highlighted in orange, while data for HMTO samples is highlighted in blue.

Differences in the reported sorption capacities in Table 4 may originate from different ratios between sorbent mass to brine volume that can influence the equilibrium state and consequently the sorption capacity. Shifts in pH values could occur faster under conditions with higher quantities of sorbent mass or smaller volumes of brine and thus may rapidly cease the ion‐exchange process (Equation (7)). In this section, the m/V ratio was varied in a range from 0.25 to 25 g L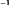 for HMO or HMTO contents of 10–1000 mg and a constant volume of 40 mL to study the influence on Li
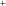
‐sorption capacity and Li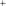 depletion in the brine. The brines contained either 1.5 or 15 g L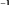 of NaOAc as buffer agent, which resulted in initial pH values of 7.4 and 8.2 in the buffered LiCl solution, respectively (as indicated by circles in **Figure** **6**c). In Figure 6a, the resulting sorption capacities (Equation (3)) based on the residual Li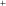 content in the LiCl solution is plotted *versus* the m/V ratio.

The second axis indicates the sorbent utilization as the ratio between *Q* 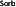 and the theoretical maximum sorption capacity *Q* 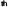 (of 68 mg g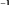) that was derived from the full conversion of H
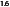
Mn
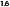
O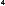 to LMO. The data in Figure 6b shows the Li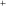 depletion as the ratio between the Li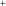 concentration in the LiCl solutions before and after the sorption experiments and Figure 6c displays the corresponding pH changes. It can be readily seen that the Li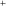 sorption is highest for the lowest m/V ratio, approaching around 5.7 mmol g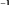 or 39 mg g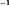 (HMTO). This value is even higher than *Q* 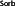 determined in the previous section by titration and corresponds to a bit over half of the theoretical sorption capacity of LMTO (57%). Although the material utilization (Figure 6a) is very high at small m/V ratios, the total amount of extracted lithium (Figure 6b) is quite small (low Li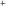 depletion) at those ratios since only a small amount of sorbent is present in a defined volume, while the volume is large enough that the exchanged protons do not change the pH significantly (Figure 6c). It is noticeable that for m/V = 0.25 the value for HMO (1.5 g L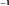 NaOAc) is significantly lower than the other values. This can likely be attributed, on the one hand, to the very low mass of sorbent at m/V = 0.25, which inherently leads to greater inaccuracies. On the other hand, the change in Li concentration is also very small at this ratio and therefore associated with a higher relative uncertainty. For this reason, the reported value was determined in five independent experiments that confirmed the result.

In contrast, at high m/V ratios, the available Li
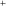
‐ion inventory per gram of sorbent is small or no longer in excess, thus generating notably smaller sorption capacities. However, with respect to a limited absolute Li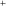 amount, the degree of Li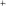 depletion is high and hence the pH changes considerable. In a batch‐type extraction process this is impractical, and hence the goal is to find an optimum balance between material utilization and extraction yield within a reasonable circulation interval. Figure 6b shows that with a concentration of 15 g L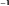 NaOAc and an sorbent content of 0.4 g HMTO almost all Li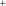 could be extracted from the LiCl solution (Li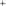 depletion of 98%). Under the same conditions the HMO sorbent material shows a Li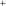 depletion of only 66%. Upon decreasing the m/V ratio of HMO further (to 1 g per 40 mL), the extraction yield approached that of HMTO, making HMTO the more economical material. The results in Figure 6b further highlight the impact of the NaOAc concentration on the extraction yield. At an initial NaOAc concentration of only 1.5 g L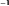, HMO and HTMO displayed extraction yields of less than 50% at an sorbent content of 400 mg. For these samples, HMO also showed somewhat higher extraction yields than HMTO.

The pH values in Figure 6c for sorption experiments with 15 g L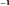 NaOAc approach a constant pH value at around 5.4 beyond an sorbent content of around 400 mg, which is well above the pK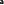 value of acetic acid (4.76) and in good agreement with the equilibrium pH of 5.6 determined from the Henderson–Hasselbalch equation.

However, with 1.5 g L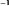 NaOAc, the pH dropped more noticeably in the sorbent mass range between 0.4 and 1 g. At an HMTO content of 1 g the pH dropped to 3.14 which is the lowest pH value in this experiment and close to the limiting pH of 2.7 measured in Figure 5. The pH change is attributed to the lower buffer capacity and suggests that the buffer is almost depleted under the conditions of this experiment. The measured pH is also lower than the theoretical value of 3.7 determined from the Henderson–Hasselbalch equation for HMTO at 1.5 g L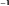 NaOAc buffer content. This may be due to the fact that the calculated pH‐change is based solely on the release of H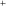 ions through ion exchange with Li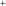 ions. It is possible that the sorbent exhibits an additional acidic effect leading to a further decrease in pH.

Compared to unbuffered systems (see previous section, Figure 5), significantly higher sorption capacities (1.5 mmol g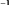, as compared to <0.5 mmol g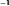 [Table 4]) could be achieved at the same m/V ratio, when the pH decrease is delayed by a buffering agent. In contrast, this approach requires sufficiently high amounts of buffering agent (NaOAc) to maintain the pH during the ion exchange.

Therefore, a possible trade‐off in this batch‐type extraction process is that it maximizes the sorbent utilization and minimizes chemical usage. In this work, this would correspond to an sorbent content of around 0.2 g per 40 mL of LiCl solution and a NaOAc concentration of 1.5 g L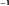, that is further used in the following experiments.

#### 4.2.3 Impact of Initial Li Content

**Figure 7:**
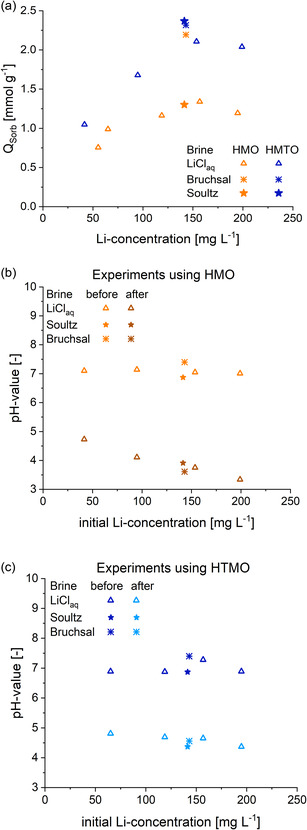
a) Sorption capacity for LiCl solutions (triangles) with various Li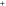 concentration and natural brines (stars). Data associated with HMO is marked orange; data for HMTO samples is marked blue. The corresponding changes in pH values before and after the sorption step are provided for b) HMO and c) HMTO.

Based on the optimized extraction conditions determined earlier (m/V = 5 and 1.5 g L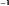 NaOAc), the sorption capacities of HMO and HMTO (triangular symbols) were determined as a function of the initial lithium concentration in buffered, synthetic LiCl solutions in the range between 40 and 200 mg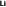 L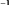 (**Figure** **7**a). In addition, the sorption capacities of the two sorbent materials in the two different buffered geothermal brines were added in Figure 7a (star‐shaped symbols). The Bruchsal geothermal brine (buffered) had a lithium concentration of 143 mg L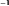 and pH of 6.3 and buffered brine from Soultz had a lithium concentration of 141 mg L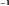 and a pH of 7.4 (Table 1). The corresponding pH changes before and after the sorption step are shown in Figure 7b,c. The sorption capacities in LiCl solutions increase approximately linearly in the range between 50 and 150 mg L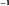, i.e., with increasing initial Li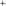 concentration. The sorption capacities obtained in solutions containing 200 mg L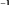 displayed slightly lower values.

As more Li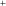 was exchanged with increasing Li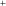 concentration, the H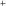 concentration increased accordingly, resulting in decreasing pH values for brines with higher Li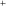 content after the sorption step.

Even though higher sorption capacities were achieved (i.e., more Li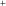 exchanged) with HMTO than HMO, the supernatant from HMTO extractions maintained a pH value near the pK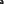 value of NaOAc/HOAc (4.76) over the tested range of LiCl concentrations. In contrast, in suspensions with HMO the pH decreased notably by ≈1.5 pH units from the lowest to the highest Li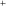 concentration.

The higher pH values in extractions conducted with HMTO favor higher sorption capacities. As a result, the sorption capacities obtained in geothermal brines with HMTO reached 2.4 mmol g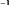 (Soultz brine) and 2.3 mmol g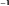 (Bruchsal brine), whereas HMO only reached similar values in sorption capacities of 2.2 mmol g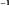 in the Bruchsal brine.

HMO displayed considerably lower sorbent capacities of only 1.3 mmol g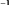, in brine from the geothermal power plant in Soultz. In addition to the initial lithium concentration, the total salinity of the Li
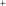
‐bearing solution (≈100 g L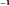, Soultz^[^ ^33^ ^]^; ≈130 g L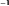, Bruchsal^[^ ^4^ ^]^) likely has an influence on the resulting sorption capacities. Interestingly, the high salinity had little impact on the pH values of the brines after the sorption process, as indicated in Figure 7b,c. Exploring these matrix effects in more detail is beyond the scope of this work, but highly relevant in developing a deeper understanding about how salinity and chemical composition of geothermal brines (at every site the brine is chemically unique) influence the ion‐exchange equilibrium.

#### 4.2.4 Ion‐Exchange Kinetics

To determine the duration of an extraction cycle and for maximized extraction yields, the ion‐exchange kinetic is a crucial factor to be considered, since in an industrial process, the time for the extraction cycle had a great impact on the size of the plant and costs. In the following sorption experiments, the sorption capacity was measured after various sorption time intervals, using the extraction conditions described earlier.

**Figure 8:**
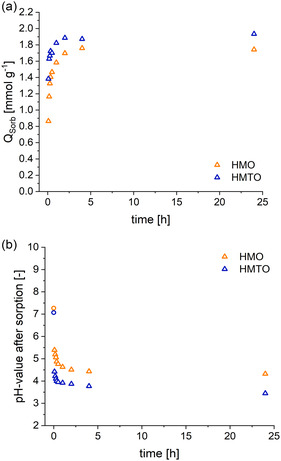
a) Sorption capacity for various reaction times and b) pH values after sorption for various time intervals are presented as triangle; the initial pH of the LiCl solution prior to sorption is shown as circles.

In **Figure** **8**a, the Li
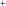
‐sorption capacity for various reaction times is plotted for HMO and HMTO. The corresponding pH values at the end of the sorption step is shown in Figure 8b. A fast ion exchange is both seen in the rapid increase of the achievable *Q* 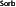 and correlated pH decrease due to the proton release into the buffered brine. HMTO reached 87% of the equilibrium sorption capacity *q* 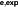 after 15 min already. By comparison, HMO only reached 76% *q* 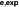 during the same time. The equilibrium value *q* 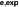 was experimentally determined after a reaction time of 24 h. Furthermore, at even smaller sorption times, the ion exchange with HMTO delivered ≈10% higher sorption capacities compared to HMO.

In accordance with the measured sorption capacities, the pH drops faster for the HMTO samples than for the HMO samples. As a result of the smaller *Q* 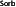 in experiments with HMO, the pH was generally higher by about 0.5–1 pH unit. As expected, the pH was lowest in both HMTO and HMO samples after the longest sorption time of 24 h, as they exhibited the highest *Q* 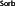 in this experiment and hence the highest proton release.

For a more quantitative understanding of the exchange kinetics, the reaction parameters were derived from a pseudo‐second‐order (Equation (2)) model. The calculated equilibrium–sorption–capacity (*q* 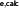) matches well with the experimental data (*q* 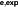), as shown in **Table** **5** and Figure S7b, Supporting Information. A better goodness‐of‐fit was found for HMO, which might be due to the fact that sorption capacities measured for HMTO at small sorption times (<10 min) displayed similar values, and hence no significant time dependency. For both sorbents, the kinetic data from the pseudo‐second‐order model is helpful in the further process design, such as reactor sizing and optimize residence time parameters for an efficient extraction process.

**Table 5:** Kinetic parameters of pseudo‐first‐order and pseudo‐second‐order kinetic models.

Sorbent	Experimental	Pseudo‐first‐order			Pseudo‐second‐order		
	*q* 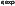	*q* 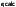	*k* 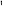	*R* 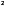	*q* 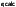	*k* 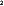	*R* 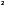
	[mmol g 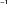 ]	[mmol g 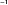 ]	[h 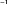 ]	–	[mmol g 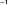 ]	[h 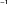 ]	–
LMO	1.74	1.67	7.18	0.89	1.76	6.56	0.99
LMTO	1.93	1.82	15.41	0.73	1.89	16.98	0.95

### 4.3 Li Selectivity and Sorbent Aging in Geothermal Brines upon Repeated Cycling

#### 4.3.1 Retention of Sorption Capacities

**Figure 9:**
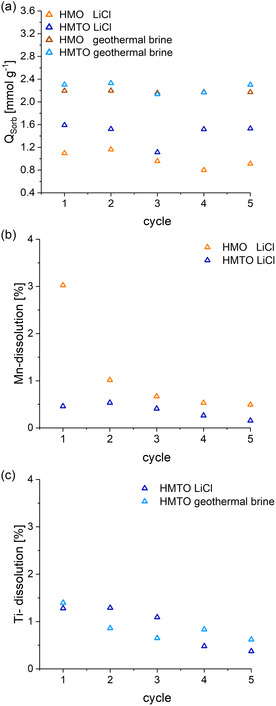
a) Sorption capacity in a cycling experiment using LiCl solution and geothermal brine. b) The Mn dissolution and c) Ti dissolution in each desorption cycle are shown.

The durability of the material over many extraction cycles is of great concern, due to potential Mn dissolution and an inhibition of Li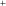 capturing due to formation of impermeable surface layers (deposits originating from competing ions). In this experiment, HMO and HMTO materials were tested with synthetic LiCl solution and Bruchsal geothermal brine over 5 sorption/desorption cycles. Based on the kinetics results, a 30 min sorption step per cycle appeared reasonable. The sorption capacities for extractions with the HMO or HMTO sorbent using LiCl solution as well as geothermal brine from Bruchsal are shown in **Figure** **9**a. Repeated Li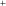 extraction was limited in this study due to the small particle sizes of the HMO and HMTO powders, which resulted in significant ion‐exchange material loss per cycle during the solid–liquid separation step. Optimizing the sorbent formulation may help mitigate material loss in this phase. This approach will be further examined in subsequent studies, potentially through the application of granulation techniques. In accordance with the kinetic experiments (t = 30 min), sorption capacities of 1.1 mmol g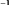 (HMO) and 1.6 mmol g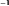 (HMTO) were measured in the first cycle using LiCl solution. In contrast, significantly higher sorption capacities were observed for the use of geothermal brine, ≈2.2 mmol g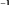 for both HMO and HMTO. While HMO and HMTO displayed constant *Q* 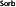 over the first 5 cycles in the geothermal brine, decreasing *Q* 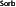 were observed in the LiCl solutions. However, HMTO recovered after the third cycle. The results for geothermal brine fit well with data from the literature, where a long‐term sorption capacity of 2.66 mmol g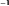 for HMO and 3.14 mmol g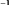 for a Mg‐substituted HMO was measured using salt lake brine.^[^ ^34^ ^]^

#### 4.3.2 Mn and Ti Leaching

A low‐manganese dissolution is attributed to a high chemical stability of the material, which is crucial for long‐term applications. In Figure 9c, the Mn dissolution over 5 cycles is presented. The cumulative Mn dissolution after 5 cycles was 5.6% for HMO and 1.8% for HMTO. The Mn losses were highest on the first cycle for HMO (2.9%) and almost constant for HMTO. The HMTO sorbent in this work showed low Mn dissolution of 0.45% in the first cycle, which is an improvement to previous studies, e.g., 3% dissolution for a Mg‐substituted HMO.^[^ ^34^ ^]^

The dissolution of Ti for the HMTO sorbent over 5 cycles is provided in Figure 9d. In the first cycle, a Ti dissolution of 1.3% (LiCl) and 1.4% (Brine) was detected. A continuous decline in the release of Ti is evident, leading to notable reduction of the Ti dissolution to only 0.4% (LiCl) and 0.6% (geothermal brine) in the 5th cycle.

Despite the dissolution of Mn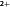 and Ti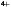, no significant reduction in sorption capacity was observed. The release of Mn could lead to the formation of vacancies or an increase in surface area, potentially enhancing the accessibility of active sites. The long‐term behavior regarding Mn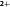 and Ti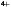 dissolution, along with its impact on sorption capacity, can be investigated in further experiments following the successful formulation of the material (e.g., granulates).

#### 4.3.3 Selectivity and Transfer Factors

The selectivity of the ion sieves HMO and HMTO was investigated by loading the materials with Bruchsal geothermal brine. Detecting the depletion of foreign ions in the brine is challenging because the concentration change for some ions, such as sodium, is very small and falls within the margin of error. Therefore, the foreign ion concentration in the desorption solution is used as a more reliable parameter in this work. As an expression for the fraction of the foreign ions that are transferred from the geothermal brine to the desorption solution through sorption at the sorbent surface or in its bulk phase, we introduce the transfer factor $T_{\text{CI}}$ (Equation (5)) as the ratio between the moles of an ion in the desorption solution and brine. A low $T_{\text{CI}}$ value is advantageous for a lithium‐enriched, high‐purity product solution. Transfer factors for the most prominent contaminants in the product solution are given in **Table** **6**.

**Table 6:** Fraction of transferred competing ions from Bruchsal geothermal brine into the desorption solution.

Sorbent	$T_{\text{Na}}$ [%]	$T_{K}$ [%]	$T_{\text{Mg}}$ [%]	$T_{\text{Ca}}$ [%]	$T_{\text{Sr}}$ [%]	$T_{\text{Ba}}$ [%]	$T_{\text{Pb}}$ [%]	$T_{\text{Fe}}$ [%]
LMO	0.02	0.11	0.15	0.21	0.54	27.1	5.3	0.37
LMTO	0.01	0.01	0.02	0.03	0.01	1.2	0.96	0.24

The results show that when using HMO and HMTO, a low percentage (<1%) of Na, K, Mg, Ca, Sr, and Fe is transferred to the desorption solution. As main constituents of the geothermal brine (Na: 36 g L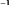, Ca: 8 g L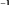, K: 3 g L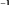, Table 1), the high Li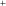 selectivity of the sorbents over these main ions is particularly important. The transfer of even small fractions of these elements would result in significant concentrations in the desorption solution, thus contaminating the Li
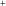
‐enriched product. Overall, HMTO proves to be superior to HMO in terms of selectivity, as fewer foreign ions are transferred.

In conventional lithium extraction Mg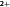 is considered challenging to separate from Li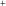, because of similar physical properties, especially their ionic radii.^[^ ^8^ ^]^ This issue did not arise in the sorption experiments conducted herein, particularly with HMTO, since only a minor amount of Mg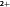 was transferred to the desorption solution.

In contrast, the separation of Li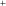 from barium ions and lead ions appears to be more challenging. HMO showed a high transfer factor of 27.1% for Ba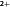 from brine to the desorption solution, that was greatly reduced to only 1.2% with HMTO. Similarly, the transfer of Pb dropped from 5.3% with HMO to 0.96% using HMTO instead. The low selectivity toward Ba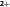 could be problematic, considering that bound barium tends to be substituted by radium, which is radioactive and present in natural brines.^[^ ^35^ ^]^ Likewise, the poor selectivity for Pb ions presents disadvantages as there are Pb isotopes in the geothermal water, which are radioactive and can lead to increased radiation levels in the sorbent.^[^ ^32^ ^]^

To assess the efficiency of lithium extraction processes, the purity of the product solution is of high importance, in addition to the lithium recovery. The composition of eluent after desorption is shown in **Table** **7**.

**Table 7:** Composition of desorption solution using HMO and HMTO as sorbent.

Sorbent	Ion concentration [mmol L 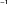 ]								
	Li	Na	K	Mg	Ca	Sr	Ba	Pb	Fe
HMO	9.03	0.31	0.09	0.02	0.43	0.02	0.02	1.3 · 10 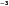	3.4 · 10 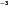
HMTO	9.1	0.13	8.5 · 10 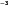	3.5 · 10 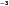	0.06	3.6 · 10 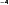	7.7 · 10 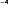	2.3 · 10 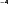	2.2 · 10 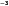

When interpreting the results, it should be noted that the composition of the product obtained under laboratory conditions (room temperature, 1 bar) may differ from that under actual geothermal conditions (20 bar, 60 °C), as the brine is only thermodynamically stable at elevated temperature and pressure. Cooling of the brine, as well as depressurization and the associated degassing of CO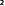, can lead to precipitation of salts. For instance, the lithium concentration in the Bruchsal brine used in this study was 143 mg L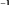, compared to 155 mg L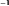 in the in situ geothermal brine. Similarly, the calcium concentration decreased from 8318 to 6900 mg L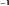 under laboratory conditions (cf. Table S3, Supporting Information, and Table 1).

In summary, the results clearly show the advantages of the HMTO sorbent. While both ion‐exchange materials are highly selective toward Li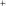 and yield similar Li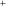 concentrations in the eluent, there is a major difference in the transfer of competing ions that are present in high contents in the brine, e.g., Na, Ca, or K. Our results demonstrate that with HMTO, a Li
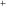
‐enriched desorption solution with smaller impurities can be achieved.

#### 4.3.4 Particle Morphologies and Growth of Surface Deposits

**Figure 10:**
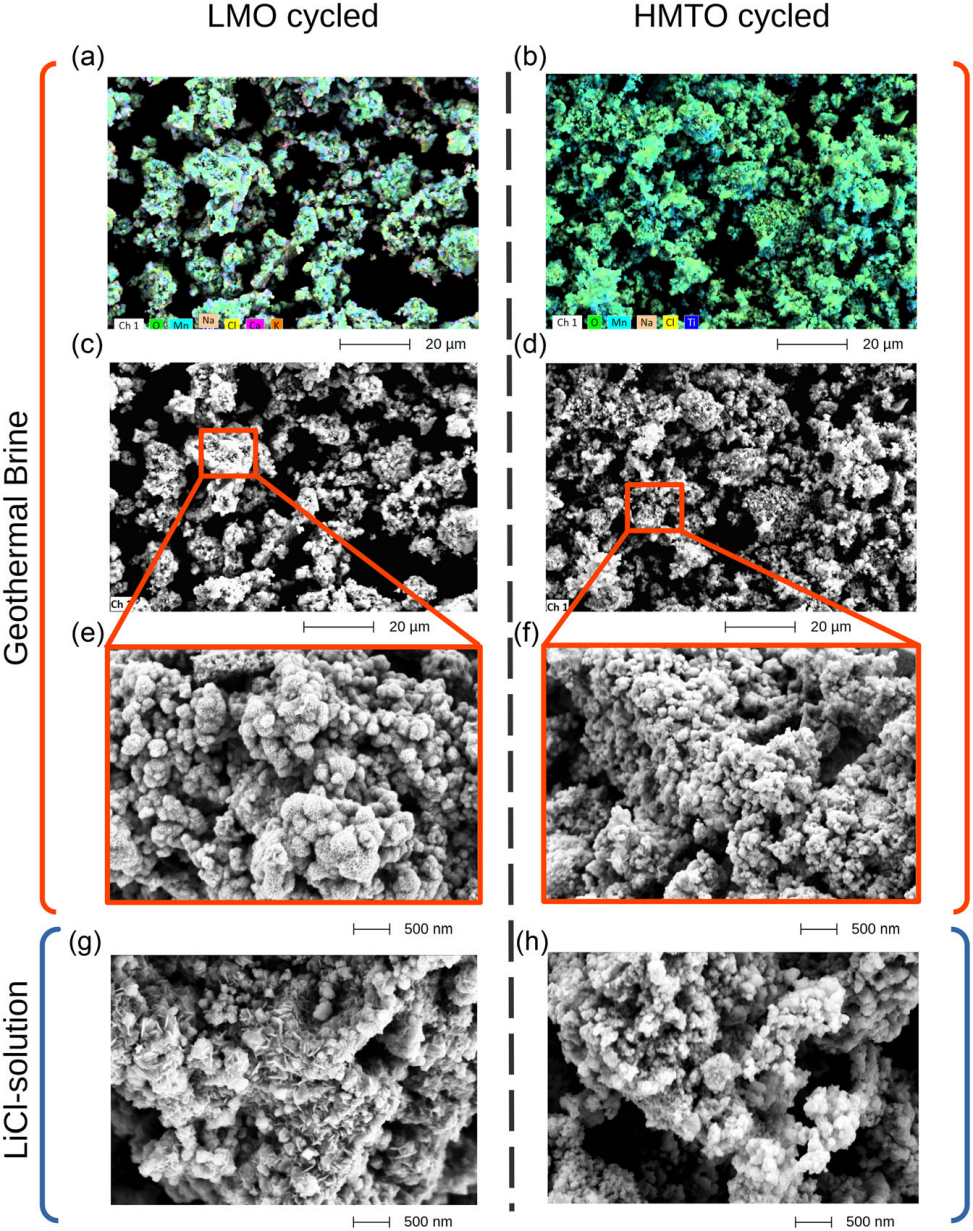
a) EDX of LMO material cycled with geothermal brine. b) EDX of HMTO material cycled with geothermal brine. c,e) SEM image of LMO after 5 sorption/desorption cycles using geothermal brine. d,f) SEM image of HMTO after 5 sorption/desorption cycles using geothermal brine. g) SEM of LMO after 5 sorption/desorption cycles using LiCl solution. h) HMTO after 5 sorption/desorption cycles using LiCl solution.

As shown in the previous section, competing ions in the geothermal brine enter the sorbent or accumulate on the surface. SEM and EDX analysis were conducted on the geothermal brine–cycled sorbents to investigate surface deposits and morphological changes over several sorption–desorption cycles. The Ti
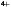
‐free material was analyzed in the Li‐loaded state (LMO), while the Ti‐substituted material was examined in the desorbed state (HMTO), each after the fifth cycle. The SEM and EDX images are shown in **Figure** **10**a,b for LMO and HMTO, respectively.

The EDX maps show mainly Mn, O, and Ti from the LMO and LMTO materials, as well as Na, Cl, Ca, and K as the main residual ions from the geothermal brine (Table 1). For cycled HMTO, the main residual ions in notable amounts were Na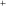 and Cl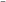, which is in general agreement with Table 6. The energy diagram (Figure S8, Supporting Information) indicates that additional minor peaks were detected for HMO and HMTO sorbents. However, these quantities are extremely low compared to the other components.

Na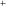 and Cl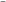 are evenly distributed over the particle surfaces, whereas the Ca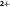 signal is concentrated on individual spots, suggesting the growth of Ca‐rich surface deposits. For comparison, in our previous study, the surface deposits on LMO sorbent synthesized *via* hydrothermal synthesis^[^ ^32^ ^]^ included also Fe, in addition to Na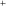, Ca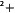, and K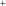 (using Bruchsal geothermal brine). The SEM images of the LMO and LMTO sorbent further suggest differences in particle sizes (Figure 10c–h). LMTO particles appear to comprise smaller primary particles than the LMO particles, which might be a reason for the faster kinetics during short sorption intervals (Figure 8). Interestingly, the comparison to LMO and LMTO particles treated only in LiCl synthetic brine indicates that the brine has a notable effect on the particle morphology of the sorbent.

#### 4.3.5 Structural Changes

Complementary to the morphological changes and the apparent loss of Mn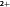 and Ti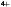 through leaching, the structural changes of the cycled Ti
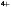
‐free LMO and HMTO were studied by XRD. The samples were prepared as described earlier. The XRD patterns are shown in Figure S10, Supporting Information.

It can be readily seen that after 5 cycles the characteristic spinel reflections are still present with slight changes in intensity. For LMO, a noticeable decrease in intensity of the reflection at 19.9° can be observed compared to the pristine LMO. When comparing the cycled substituted materials with the pristine ones, it is evident that the reflection intensities and positions are still well maintained in the Ti‐substituted samples, suggesting high structural stability. Generally, for cycled sorbents, there is an observable trend of reflections becoming narrower compared to pristine materials. This phenomenon may be attributed to the loss of fine (nano‐)crystallites during the separation process in the cycling experiments. This observation is further supported by the SEM images. The gradual loss of material currently still hampers long‐term cycling and stability tests.

## 5 Conclusion

In this study, we introduced a novel spray‐drying approach as an enabler for facile upscaling of LMOs for DLE applications. Therefore, our spray‐drying approach helps to bridge the existing gap in material availability of LMO‐based ion‐exchange materials and can facilitate implementation on the pilot scale. It was further demonstrated that this approach allowed to readily substitute small fractions of Mn by other transition metals, here Ti, to produce mixed lithium transition metal oxides to further tailor the material properties. The LMO with composition Li
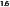
Mn
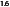
O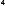 and LMTO with composition Li
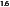
Ti
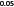
Mn
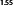
O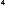 were applied to extract Li from geothermal brines and synthetic LiCl solutions with similar Li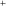 concentrations. In this context, the method represents a significant step forward to an industrial scale extraction of lithium as the maximum sorption capacities of 5.05 mmol g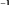 (35.05 mg g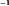) for HMO and 5.66 mmol g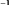 (39.28 mg g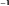) for HMTO under ideal m/V ratios show a considerable improvement to previous studies on Mn‐based sorbents from hydrothermal synthesis approaches.^[^ ^30^, ^32^ ^]^ This was also reflected in lower Mn‐dissolution rates on the first extraction and recovery cycle for HMO produced from the spray‐drying approach.^[^ ^32^ ^]^ Further improvements in terms of dissolution rates were achieved through Ti substitution, resulting in Mn‐dissolution rates of <0.5%, which is likely attributed to the stabilizing effect of Ti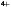 in the crystal structure. Especially on short time scales, HMTO also exhibited faster ion‐exchange kinetics. Furthermore, the m/V experiments demonstrated that compared to HMO smaller amounts of HMTO are required to extract equivalent amounts of lithium. Selectivity studies further showed that using HMTO sorbent achieved higher product purity compared to HMO. The combination of these properties make HMTO overall, more favorable, with respect to an industrial extraction process. The loss of sorbent mass on every separation step currently limits the feasible number of extraction cycles that can be performed with the batch‐type extraction setup used herein and will be addressed in future work.

## Conflict of Interest

The authors declare no conflict of interest.

## Supporting information

Supplementary Material

## Data Availability

The data that support the findings of this study are available from the corresponding author upon reasonable request.
