# Supplementary material for: Synthesis of Lithium Manganese Oxide and Ti‐Substituted LMO Sorbents for Lithium Extraction in a Spray‐Drying Process
Source: ChemSusChem. 2025 May 23;18(13):e202500530. doi: 10.1002/cssc.202500530 (PMC12232121; doi:10.1002/cssc.202500530)
Supplement: Supplementary file 1 — Supplementary Material [file CSSC-18-e202500530-s001.pdf]

Synthesis of Lithium manganese oxide (LMO) and Ti-Substituted LMO Sorbents (LMTO) for Lithium Extraction in a Spray Drying Process

Laura Herrmann,[a,b]* Nicole Bohn,[a] Alisa Pfau,[b] Thomas Kölbel,[b] Helmut Ehrenberg,[a] Joachim Binder,[a] Fabian Jeschull,*[a]

[a] L. Herrmann, N. Bohn, Prof. Dr. H. Ehrenberg, Dr. J. Binder, Dr. F. Jeschull*
Institute for Applied Materials - Energy Storage Systems (IAM-ESS), Karlsruhe Institute of Technology - Hermann-von-Helmholtz-Platz 1 D-76344 Eggenstein-Leopoldshafen
E-mail: fabian.jeschull@kit.edu

[b] L. Herrmann*, A. Pfau, Dr. T. Kölbel
R&D Department - EnBW Energie Baden-Württemberg AG, Durlacher Allee 93, 76131 Karlsruhe
E-mail: l.herrmann@enbw.com

Supplementary Material

BET-Parameters The original BET equation, named after Brunauer, Emmett, and Teller, is an empirical equation used to determine the specific surface area of sorbents (1). It is given by:

$$\frac{p}{n^a(p_0 - p)} = \frac{1}{n^a_m \cdot C} + \frac{C-1}{C} \cdot \frac{p}{p_0} \cdot \frac{1}{n^a_m} \tag{1}$$

Where n^a is the amount of adsorbed at the relative pressure p/p_0 and n^a_m is the monolayer capacity. In accordance with the BET theory, the parameter C exhibits an exponential relationship with the enthalpy (heat) of sorption within the first adsorbed layer. A molecular cross-sectional area a_m of 0.1620 nm^2 was considered.

Table S1: Parameters of BET Analysis

Sample	n_m^a mmol g^{-1}	C -	p/ p_0 -	Y-Intersept g mmol^{-1}	Sat. pressure mmHg
LMO-800	0.0143	139.657	0.2916	0.501 ± 0.026	777.96
LMO-720	0.0297	110.843	0.2921	0.303 ± 0.006	764.44
LMO-720-450	0.0231	136.290	0.2918	0.317 ± 0.015	777.96
LMO-450	0.0265	122.212	0.2915	0.308 ± 0.008	777.96
LMTO-450	0.0652	133.945	0.2913	0.114 ± 0.006	761.84

The isotherm linear plots and the BET surface area plots for LMO materials calcined at different temperatures and LMTO are shown in Fig. S1 and S2.

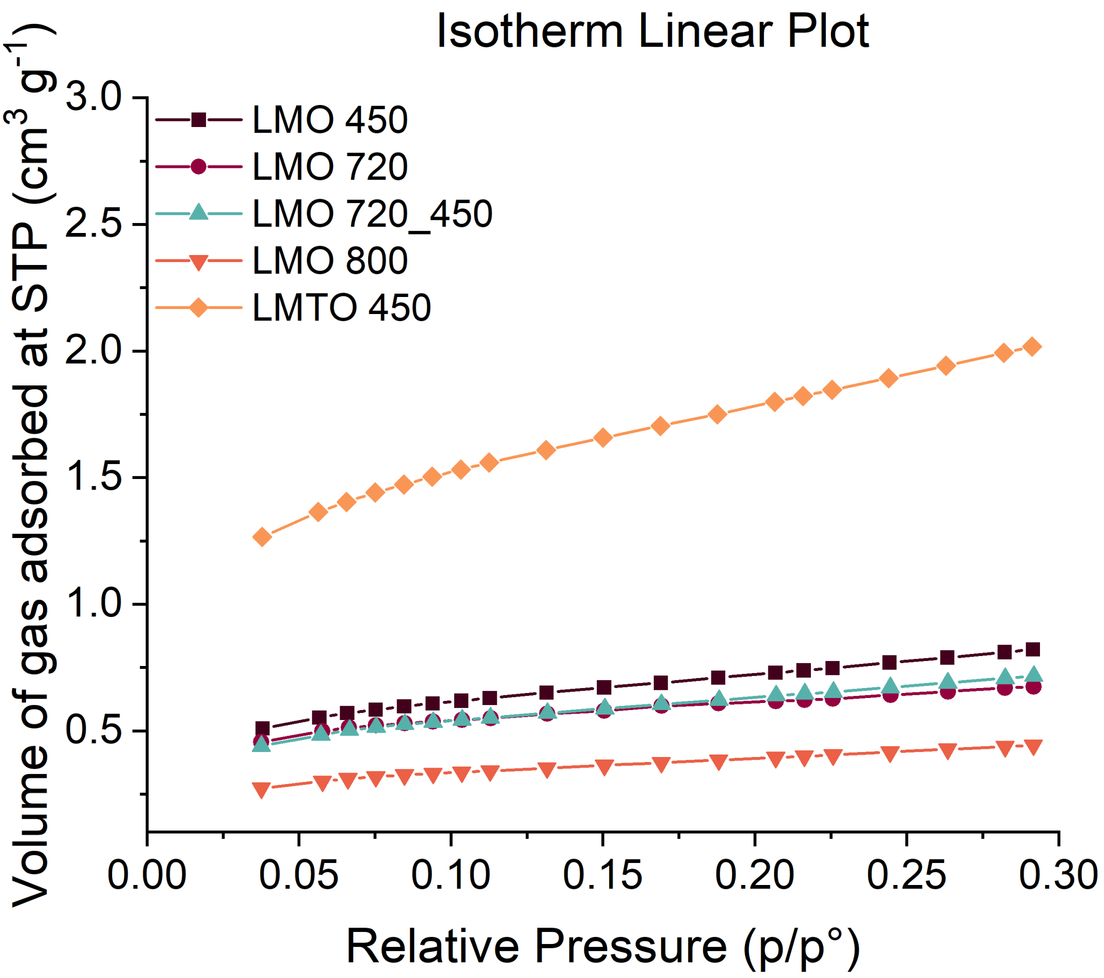

Figure S1: BET: Isotherm Linear Plot

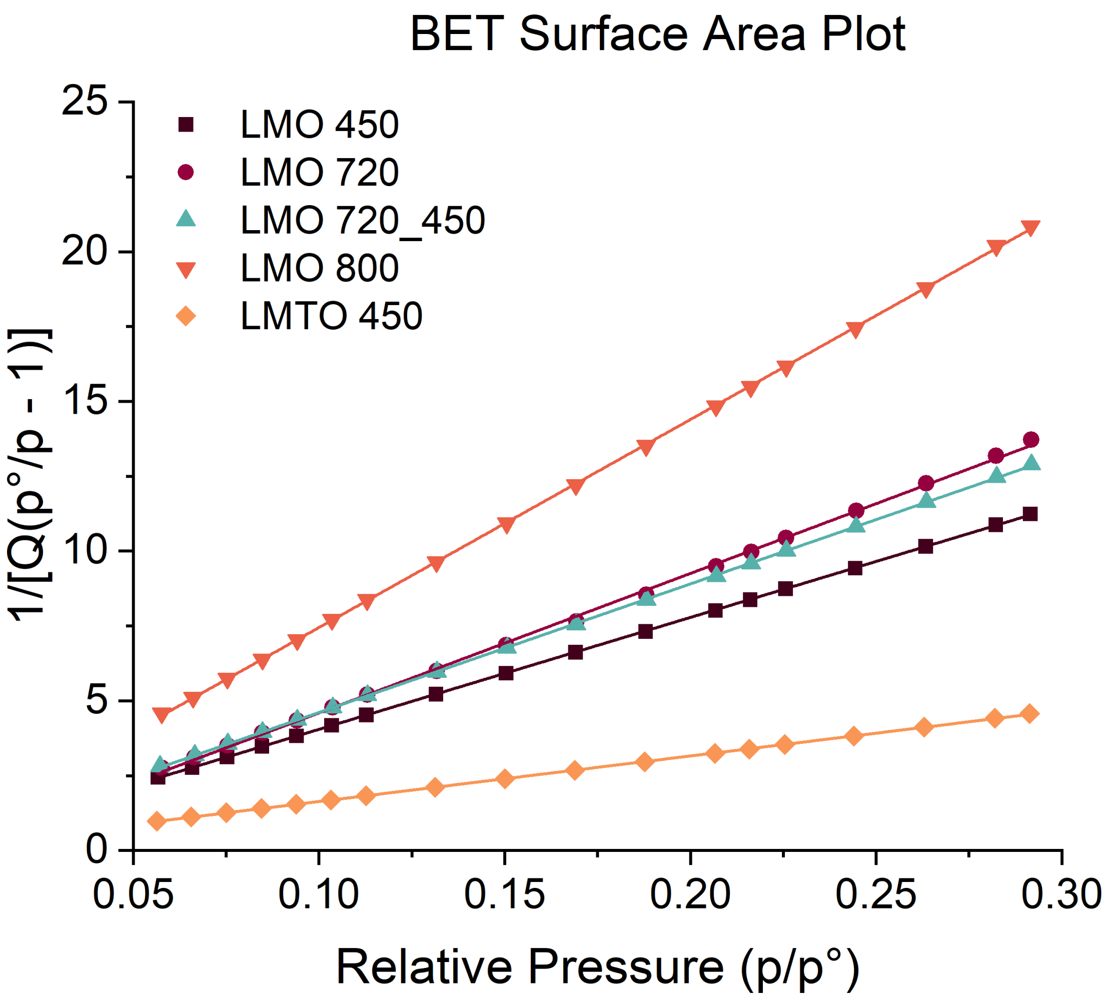

Figure S2: BET: Surface Area Plot

Ti^{4+} substitution Fig. S3a shows the diffractograms of the Li-Mn-Ti precursor, the calcined LMTO and activated HMTO. Fig. S3b and c show SEM images of the pristine LMTO and HMTO. Fig. S3d-f show the elemental distribution of the LMTO powder.

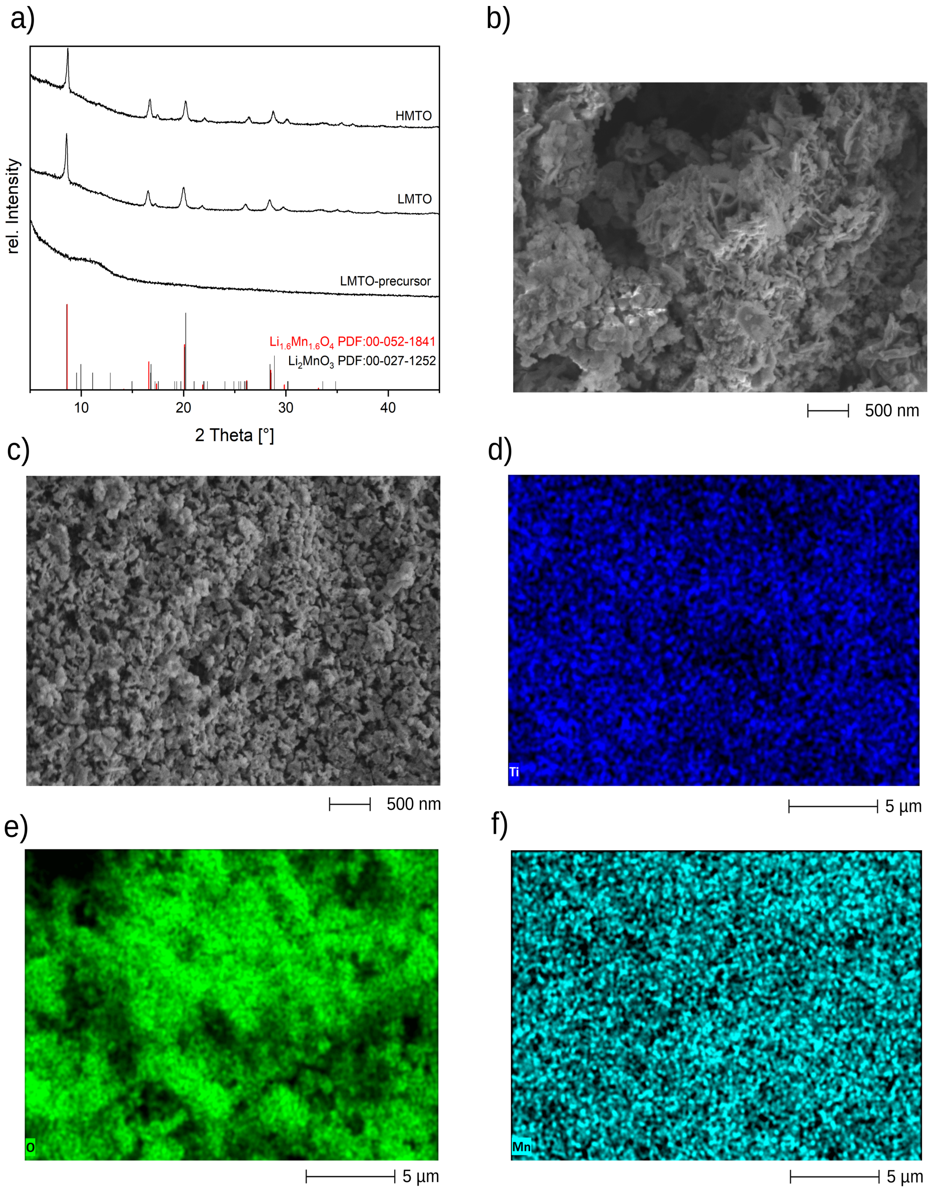

Figure S3: a) X-ray diffraction patterns of the LMTO precursor, pristine LMTO and activated HMTO. SEM images of the as-prepared LMTO and activated HMTO powders are shown in b) and c), respectively. The elemental distribution in the LMTO powder is shown for Ti (d), O (e) and Mn (f)

TGA

A TGA of the Ti-substituted LMTO precursor was conducted. The results are shown in Fig. S4.

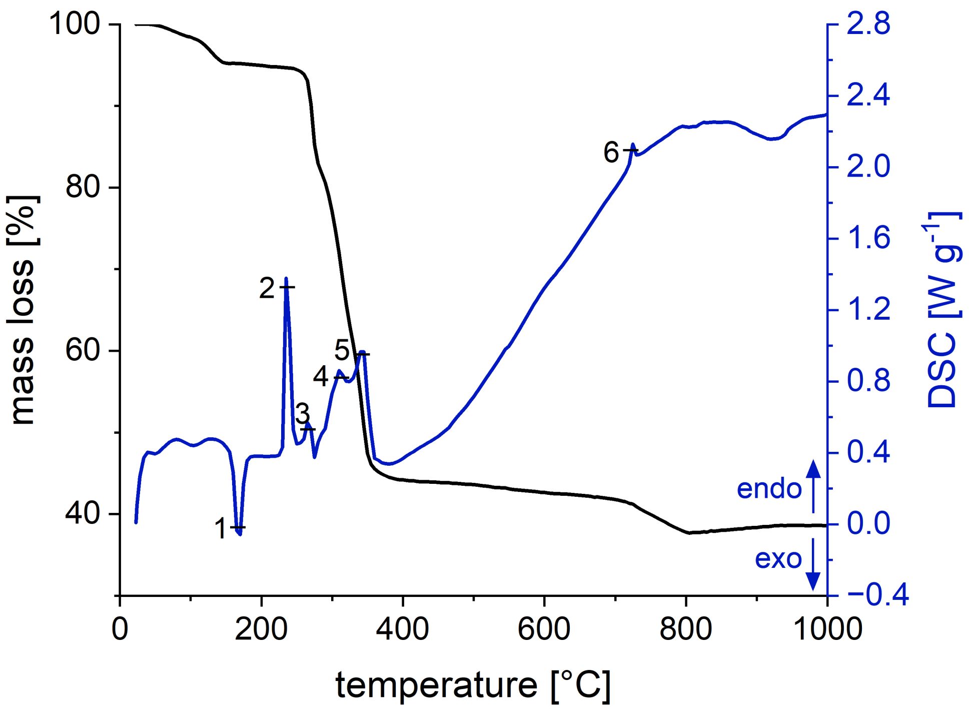

Figure S4: TGA of LMTO

Table S2: TGA: Significant Peaks

Peak	T [°C]	Reaction Type
1	167	exothermic
2	237	endothermic
3	268	endothermic
4	311	endothermic
5	343	endothermic
6	727	endothermic

Geothermal Brine

The compositions of the geothermal brines used in this study are presented in Tab. S3.

Table S3: Compositions of the geothermal brines used in this study

	c	Li	Na	K	Mg	Ca	Sr	Ba	Pb	Al
Bruchsal	[mg L^{-1}]	143	35700	3091	382	8318	391	8.9	4.9	1.9
	[mmol L^{-1}]	20.6	1552	79.1	15.7	207	4.5	0.07	0.02	0.07
Soultz	[mg L^{-1}]	141	27870	3430	125	7443	479	6.2	0.3	0.5
	[mmol L^{-1}]	20.4	1212	87.8	5.2	186	5.5	0.05	0.001	0.02

Rietveld Refinement

The XRD-data was collected using X-ray diffraction analysis (D8 Advance, Bruker) with Cu K_α radiation source. A Rietveld Refinement was conducted using FullProf Software for LMO calcined at 450 °C and 800 °C. For LMO-450°C, a single-phase spinel structure was assumed, while for LMO-800°C, both a spinel structure and a layered Li_2MnO_3 impurity phase were considered. The initial structural models for the spinel phase (space group $Fd\bar{3}m$) and the layered Li_2MnO_3 impurity phase (space group C2/m) were obtained from the ICDD database. The refinement results showed a χ^2 value of 3.2 for LMO-450°C and 2.2 for LMO-800°C. The results of the fitting are shown in Fig. S5.

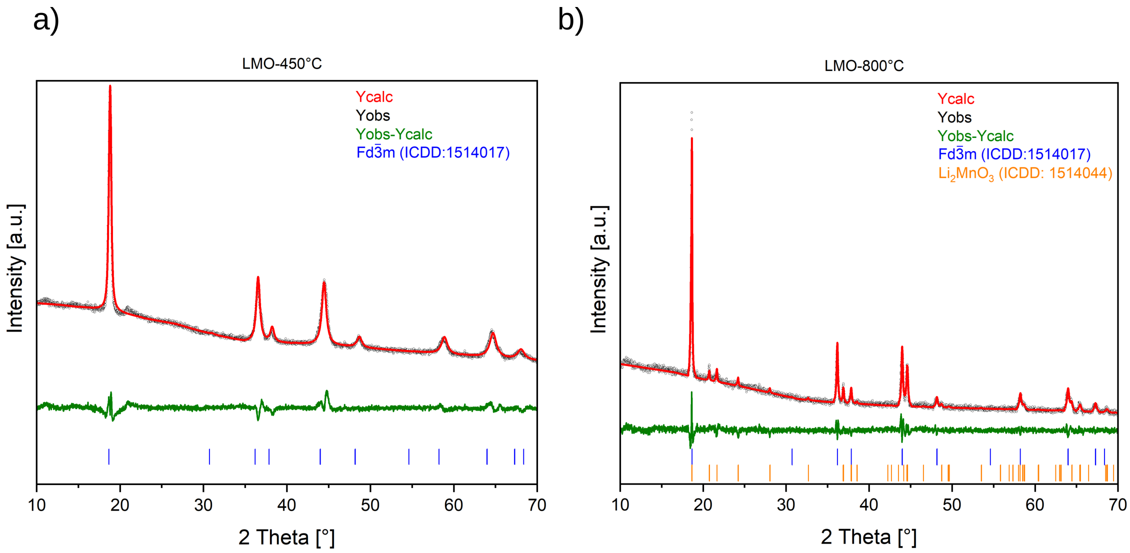

Figure S5: Rietveld Refinement of LMO calcined at 450 °C (a) and 800 °C (b)

pH value after sorption in a buffered system The calculation of the expected pH value after sorption is demonstrated in the following example. A LiCl solution with 150 ppm Li was prepared, and NaOAc at a concentration of 1.5 g/l (corresponding to approximately 0.018 mol/l) was added. The initial pH is 7.41. The pK_a of acetic acid (HA) is approximately 4.76. The ratio of $[A^-]$ (acetate ion) to [HA] (acetic acid) can be determined using the Henderson-Hasselbalch equation:

$$\mathrm{pH} = \mathrm{p}K_a + \log\left(\frac{[\mathrm{A}^-]}{[\mathrm{HA}]}\right)$$

By substituting the values, we get:

$$7.41 = 4.76 + \log\left(\frac{[\mathrm{A}^-]}{[\mathrm{HA}]}\right)$$

$$\log\left(\frac{[\mathrm{A}^-]}{[\mathrm{HA}]}\right) = 7.41 - 4.76 = 2.65$$

$$\frac{[\mathrm{A}^-]}{[\mathrm{HA}]} = 10^{2.65} \approx 446.68$$

By rearranging, we get:

$$[\mathrm{HA}] = \frac{[\mathrm{A}^-]}{446.68}$$

For 40 mL (0.04 l) of the solution, the molar amount of NaOAc is:

$$n(\mathrm{A}^-) = 0.018\ \mathrm{mol/l} \times 0.04\ \mathrm{l} = 7.2 \times 10^{-4}\ \mathrm{mol}$$

The molar amount of acetic acid ist (n(HA)) is:

$$n(\mathrm{HA}) = \frac{7.2 \times 10^{-4}\ \mathrm{mol}}{446.68} \approx 1.61 \times 10^{-6}\ \mathrm{mol}$$

During the sorption step, an ion exchange of Li^+ and H^+ ions occurs. In this experiment a release of 0.673 mmol H^+ was observed (*Fig. 7a*), which results in:

$$n(\mathrm{A}^-)_{\mathrm{neu}} = 7.2 \times 10^{-4}\ \mathrm{mol} - 6.73 \times 10^{-4}\ \mathrm{mol} = 5.9 \times 10^{-5}\ \mathrm{mol}$$

$$n(\mathrm{HA})_{\mathrm{neu}} = 1.61 \times 10^{-6}\ \mathrm{mol} + 6.73 \times 10^{-4}\ \mathrm{mol} = 6.75 \times 10^{-4}\ \mathrm{mol}$$

New concentrations result, as shown below:

$$[\mathrm{A}^-]_{\mathrm{neu}} = \frac{5.9 \times 10^{-5}\ \mathrm{mol}}{0.04\ \mathrm{l}} = 14.75 \times 10^{-4}\ \mathrm{mol/l}$$

$$[\mathrm{HA}]_{\mathrm{neu}} = \frac{6.75 \times 10^{-4}\ \mathrm{mol}}{0.04\ \mathrm{l}} = 168.75 \times 10^{-4}\ \mathrm{mol/l}$$

By substituting the values into the Henderson-Hasselbalch equation, we get:

$$pH_{neu} = pK_a + \log\left(\frac{[A^-]_{neu}}{[HA]_{neu}}\right)$$

$$pH_{neu} = 4.76 + \log\left(\frac{14.75 \times 10^{-4}}{168.75 \times 10^{-4}}\right)$$

$$pH_{neu} = 4.76 - 1.06 = 3.7$$

The expected pH value due to the ion exchange is 3.7.

Fitting kinetic models The Python code used for kinetic fitting is shown in Figure S6.

```python

import numpy as np
from scipy.optimize import curve_fit
import matplotlib.pyplot as plt
from sklearn.metrics import r2_score


def first_order(t, q_e, k_1):
    return q_e * (1 - np.exp(-k_1 * t))

def second_order(t, q_e, k_1):
    return t / ((1 / (k_1 * q_e * q_e)) + t / q_e)

t = np.array([5/60, 1/6, 0.25, 1/3, 0.5, 1, 2, 4, 24])
data = [
    {
        'name': 'LMO',
        'qt': np.array([0.86344, 1.16493, 1.326512968, 1.40567, 1.465763689, 1.58286, 1.697636888,1.759913545,1.742420749]),
        'qt_errors': np.array([0.246973, 0.365481, 0.370584598,0.2987643, 0.314758807, 0.2456782, 0.317932795,0.230323481,0.194741936])
    },
    {
        'name': 'LMTO',
        'qt': np.array([1.3837, 1.6291, 1.667896254, 1.72346, 1.700417867, 1.8233, 1.885677233, 1.871080692, 1.933962536]),
        'qt_errors': np.array([0.13567, 0.11482, 0.137307926, 0.12567, 0.121903305, 0.11473, 0.115005228, 0.117812957, 0.113148441])
    }
]
orders = [{'name': 'first', 'method': first_order}, {'name': 'second', 'method': second_order}]

for material in data:
    for order in orders:
        popt, pcov = curve_fit(order['method'], t, material['qt'], sigma=material['qt_errors'], absolute_sigma=False)
        q_e, k_1 = popt
        q_e_error, k_1_error = np.sqrt(np.diag(pcov))
        label = f"Original data {material['name']} {order['name']} order with absolut_sigma=false"
        t_fit = np.linspace(min(t), max(t), 100)
        qt_fit = order['method'](t_fit, q_e, k_1)

        r2 = r2_score(material['qt'], order['method'](t, q_e, k_1))

        with open("results.txt", "a") as file:
            file.write(f"{label}\n")
            file.write(f"q_e = {q_e} +/- {q_e_error}\n")
            file.write(f"k_1 = {k_1} +/- {k_1_error}\n")
            file.write(f"R^2 = {r2}\n")
            file.write("\n")

        plt.figure()
        plt.errorbar(t, material['qt'], yerr=material['qt_errors'], fmt='o', label=label)
        plt.plot(t_fit, qt_fit, 'r', label='Fitted curve')
        plt.xlabel('Time t (h)')
        plt.ylabel('q_t (mmol/g)')
        plt.legend()
        plt.savefig(f"{label}.png", dpi=300)
```

Figure S6: Python Code: Fitting of kinetic model pseudo first and pseudo second order.

The Fitting results are presented in Figure S7

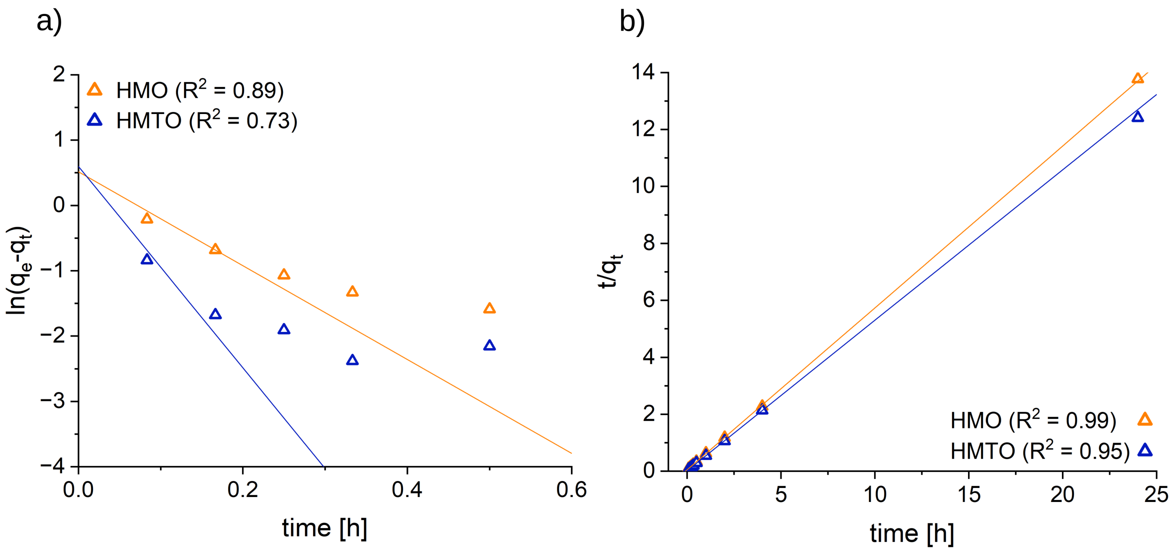

Figure S7: a) Fitting of k_1 and $q_{e,calc}$ using pseudo-first-order kinetic model. b) Fitting of k_2 and $q_{e,calc}$ using pseudo-second-order kinetic model.

EDX-Energy Diagram The Energy Diagram of the HMO and HMTO EDX-Analysis is shown in Fig. S8.

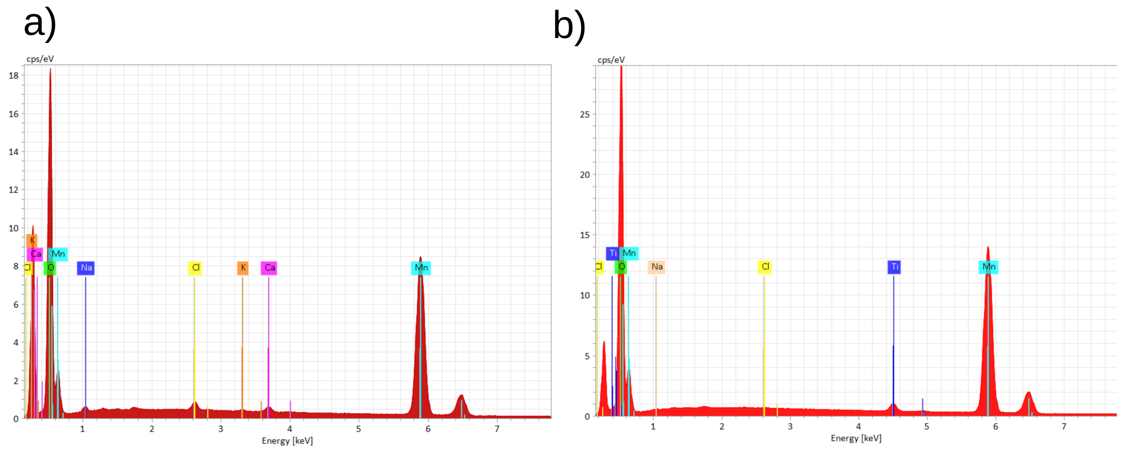

Figure S8: Energy diagram of sorbent cycled with geothermal brine a) HMO and b) HMTO

Particle Size distribution (PGV) An analysis was conducted to determine the particle size distribution of the precursor, pristine LMO, and activated HMO. The Data is shown in Fig. S9.

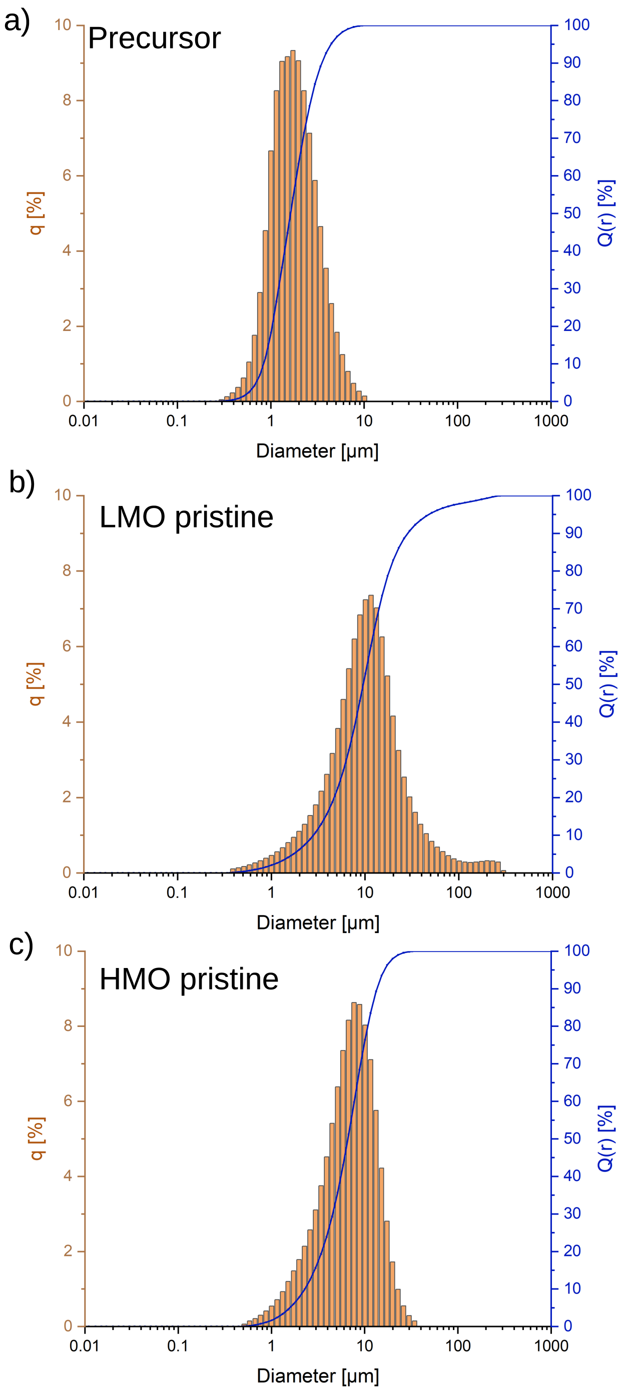

Figure S9: PGV of the precursor (a) , sorbent material LMO (b), and activated material HMO (C).

Structural changes of sorbent after cycling

The structural changes of the cycled Ti^{4+}-free LMO and Ti-substituted HMTO were studied by XRD. The diffractograms are shown in Fig. S10.

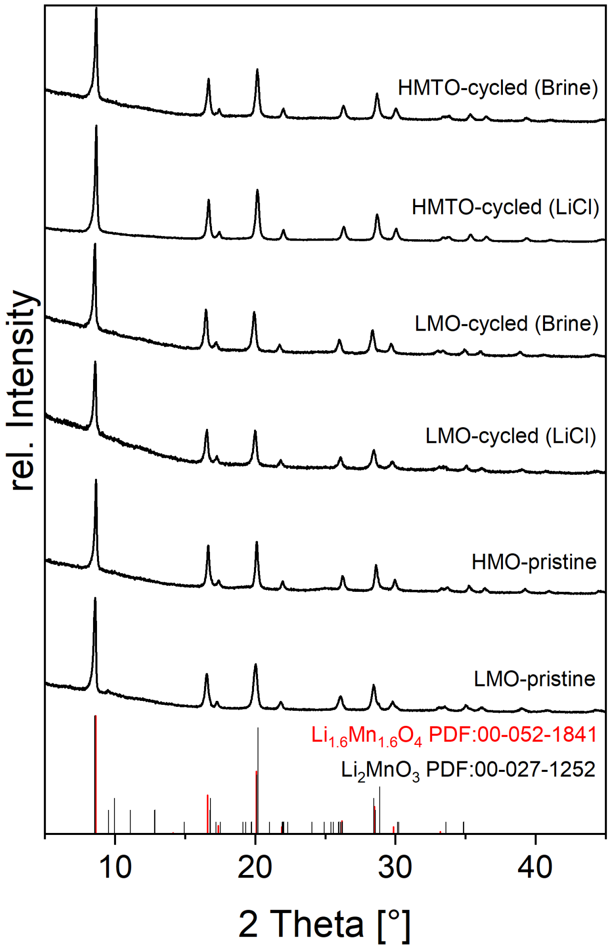

Figure S10: a) X-ray diffraction patterns of pristine and cycled LMO and HMTO using LiCl-solution or geothermal brine after 5 sorption/desorption cycles.

References

K. S. W. Sing, “Reporting physisorption data for gas/solid systems with special reference to the determination of surface area and porosity (recommendations 1984),” *Pure and Applied Chemistry*, vol. 57, no. 4, pp. 603–619, 1985.
